# Similarities and differences between dog–human and human–human relationships

**DOI:** 10.1038/s41598-025-95515-8

**Published:** 2025-04-22

**Authors:** Borbála Turcsán, Dorottya Júlia Ujfalussy, Andrea Kerepesi, Ádám Miklósi, Enikő Kubinyi

**Affiliations:** 1 Department of Ethology, ELTE Eötvös Loránd University, Budapest, Hungary; 2 MTA-ELTE Lendület “Momentum” Companion Animal Research Group, Budapest, Hungary; 3 ELTE NAP Canine Brain Group, Budapest, Hungary; 4 ELTE NAP Comparative Ethology Research Group, Budapest, Hungary

**Keywords:** Dog-owner relationship, Social support, Social network, Comparative analysis, Relationship dynamics, Psychology, Human behaviour, Social evolution

## Abstract

Most dog owners in Western countries regard their dogs as companions or family members, prompting extensive research on the human-animal bond. However, many studies rely on broad categories or single dimensions, such as satisfaction or attachment, which can oversimplify the complexity of these relationships. In contrast, our study employed a multifaceted approach, examining the dog-owner bond across 13 relationship scales and comparing these scales to four different types of human relationships to reveal the human-like roles of dogs within a family. Participants (*N* = 717) characterized their relationships with their dog and four human partners: closest kin, romantic partner, best friend, and child, using the Network of Relationships Inventory. The results showed that owners reported greater satisfaction with their dogs than with any human partner except their child. They received more Support from dogs than from any human partner except their child and experienced fewer Negative Interactions with their dogs compared to any human partner, except their best friend. Overall, the relationship with the dog provided high Companionship, opportunities for Nurturance, and minimal Negative Interactions. This may stem from the fact that the dog-human relationship features a more asymmetric power dynamic than human relationships – i.e., owners have full control over the dog’s life. Interestingly, a good relationship with a dog might also reflect the owner’s positive relationships with humans. In conclusion, the dog-human relationship exhibits characteristics of both child and best friend relationships, combining the positive aspects of the parent-child bond with the minimal negative interactions of close friendships. Characterizing the dog-owner relationship as a set of social provisions offers a valid way to evaluate relationships with dogs, providing an alternative theoretical framework to attachment theory for comparing dog-human and human-human relationships.

**Supplementary Information:**

The online version contains supplementary material available at 10.1038/s41598-025-95515-8.

## Introduction

Dogs have been a part of human society longer than any other domesticated species, but their roles have evolved significantly over time. Initially, dogs served various practical functions and lived alongside humans as pariah or free-ranging dogs; these village dogs still constitute the majority of the global dog population today^1^. However, there is also evidence of dogs being kept for sentimental reasons from the very beginning^2^. Over millennia, the proportion of companion dogs increased, and our relationship with them evolved in response to changes in human society and needs. By the 19th century, dogs were referred to as “man’s best friend,” indicating their value beyond practical use, being appreciated for their loyalty and companionship. By the 1980s, studies in the USA reported that the majority of dog owners considered their pets integral members of the family^3,4^. More recently, dogs have come to be viewed as child substitutes, as evidenced by terms such as “pet parents” and “fur baby”^5^, and the establishment of Pet Parent’s Day in 2007 by Veterinary Pet Insurance (https://www.nationaldaystoday.com/national-pet-parents-day/).

The primary theoretical framework used to explain the human-dog relationship is attachment theory^6^, specifically the child-parent attachment. Attachment encompasses four sub-behaviour systems: proximity maintenance, separation distress, safe haven, and secure base, which help maintain safety in stressful situations. Dogs exhibit all four behaviour systems^7,8^, and, in addition, the dog-human relationship shares many similarities with child-parent relationships in aspects such as power balance, dependence, nurturance, and teaching^9^.

However, there are notable differences. For instance, the dog-owner attachment could be seen as more reciprocal than the child-caregiver bond^9^ because dogs often serve as attachment figures for their owners^10–12^. Dogs also fulfil different roles compared to children. Some people seek companionship and entertainment, while others need consistency, trust, and unconditional acceptance from their dogs, resembling adult attachment relationships with friends or romantic partners^11,13^. Consequently, Savalli and Mariti^9^ suggest combining friendship and child-caregiver attachment theories to fully explain the human-dog relationship.

While some dogs are still kept solely for protection or work, in Western societies, most have lost a specific affordance but form a significant bond with their owners. However, the role dogs play in the social network of their owners may not be constant but may depend on the life stages of the owner. Turner^14^ identified six stages of the family life cycle during which pets fulfil different roles. In the early stages, pets serve as companions for independent young adults, then as child precursors or substitutes for newlywed couples^15,16^. As families grow, pets become companions or sibling-substitutes for children, and later, the first pet (and responsibility) of adolescents, who often take on their care^17^. In later stages, such as the empty nest and family reorganization, pets may once again play a substitute role, helping cope with potential losses and mitigating loneliness. This theoretical overview is also supported by empirical research. For example, owner age has been shown to affect how and from where owners acquire a dog^18^, as well as what characteristics they consider important when selecting a dog^19^. Studies have also shown that owners with children engage less in shared activities with their dogs, consider them less as friends, and spend less time and money on their care^20,21^. Attachment to pets is stronger in newlyweds, singles, and empty-nesters, likely due to limited support and affection from other sources^3,22^.

Many studies attempt to quantify dogs’ roles in their owners’ lives and categorize these relationships. Typically, owners are asked to select categories such as ‘family member,’ ‘child,’ ‘pet,’ or ‘colleague’^20,23–26^. However, these categories lack clear definitions and real-life relevance. For instance, when owners report that they consider their dogs to be children, it does not necessarily mean the dogs fulfil all child roles or are treated exactly like children. These categories might serve only as labels, and their use could be strongly influenced by (social) media trends rather than reflecting actual relationships. Moreover, while studies predominantly report a very high proportion of dogs regarded as ‘family members’, this designation could mean anything from newborn to distant cousin, providing little insight into the actual quality of the relationship. Finally, treating dog ownership as a simple categorical attribute can be misleading, as individuals are unlikely to be entirely consistent in their attitudes and actions toward the dog^27^.

Describing the role of dogs with a single category oversimplifies the complex dog-owner relationship. Even studies using quantitative measures often rely on single continuums, such as general satisfaction (e.g^28^). , or the level of attachment to the dog^29–31^. A single scale expressing love or satisfaction cannot fully convey a dog’s role in an owner’s life^32^. However, the most significant issue is that these evaluations apply only to animals and cannot be integrated into any human relationship framework. Consequently, they offer little in terms of placing the animal within the human social network.

Although the attachment theory can be applied to both human and dog relationships, allowing for a direct comparison between them^33^, there are aspects of the relationship that are not captured by this framework. Moreover, most relationship scales measuring the degree of human ‘attachment’ to companion animals are not fully congruent with the attachment theory^34^, as they primarily reflect the degree of affection, emotional closeness, companionship, and shared activities^35^. To better understand a dog’s place in our social network, we could explore the forms of emotional and social support they provide. Our social network includes various partners (e.g., parents, friends, colleagues) who offer different types of support (e.g., admiration, consistency, aid, nurturance). Relationships can be characterized by patterns of these features, such as the level of companionship, conflict, or power dynamics (e.g., who makes decisions in a relationship). Many characteristics describing human relationships also apply to human-dog relationships^3,36^, which could offer an alternative framework for studying the dog-human relationship in a directly comparable way. However, to date, only one study has investigated the characteristic features of the pet-human relationship within this comparative framework. Bonas et al.^37^ used the Network of Relationships Inventory (NRI^38^), on a sample of owners from the UK to compare human and pet relationships. They found that participants received more social support from human relationships but reported less conflict with pets. Pet dogs scored higher on Companionship, Nurturance, and Reliable Alliance scales compared to humans, while cats rivalled humans in Nurturance and Reliable Alliance. Human partners excelled only in Instrumental Aid and Intimacy. Despite promising results, this pioneering study could benefit from methodological improvements in the following areas:

1. **Validity of the scales**: The thirteen relationship characteristics of the inventory were not validated for pet-human (dog-human) relationships, only for the super-scales.
2. **Comparisons**: The authors combined the ratings of various human partners into a single “human-human relationship” category and, in many comparisons, also merged different pet species into a single “human-pet relationship” category. This approach may have masked important differences, as different human relationships can exhibit distinct patterns^39^, and dogs and cats differ in many relationship traits^32^.
3. **Statistical limitations**: The study had a small sample size (90 participants) who provided ratings for 244 human-pet relationships. However, it treated multiple pets from the same owner as independent data points and used independent sample tests instead of paired tests to compare human-human and human-pet relationships.

Thus, our study aims to replicate and complement the Bonas et al.^37^ study by investigating the qualitative and quantitative features of the dog-human relationship in greater detail and with enhanced statistical rigor. We have three specific research questions:

1. **Relationship characteristics**: Can the relationship with a dog be characterized by the same qualities used to describe human relationships? We conduct a factor analysis on dog relationship items to validate whether the same relationship characteristics can be applied to dog-human relationships.
2. **Comparative evaluation**: How do owners evaluate their relationship with their dogs compared to human relationships? We replicate the Bonas et al.^37^ study by comparing dog-human relationships to various human-human relationships using paired statistics. Additionally, we extend the scope of the study by including human partners outside the family of origin (children, romantic partners, best friends) and analysing not only individual scales but also combinations, to determine whether the dog-owner relationship exhibits a unique pattern or matches a specific type of human relationship. Moreover, we investigate potential correlations between the ratings of dogs and human partners, which would suggest that owners may use the dog to compensate for the insufficient support in their human relationships. We hypothesize:
  i. Owners perceive less conflict with dogs and rate Companionship, Nurturance, and Reliable Alliance higher for dogs than for human partners (with the possible exception of the parent-child relationship), based on the results of Bonas et al.^37^.
  ii. The overall pattern of the human-dog relationship qualities will most closely resemble the parent-child relationship, based on their shared characteristics, such as an asymmetrical power balance, dependence, nurturance, and teaching. Additionally, the human-dog relationship will be more similar to actively chosen relationships (romantic partners, friends) than to kin (genetic) relationships, with the exception of the parent-child relationship.
  iii. We expect no correlation or a positive correlation between human-human and human-dog support, similar to^37,40,41^.
3. **Influence of factors**: How do different factors influence the dog-human relationship characteristics? Since the life stages of the owner, especially parenthood and age, affect the role the dog plays in the owner’s life^14^, one could expect that these factors also influence the characteristics of the dog-human relationship. Similarly, the dog’s age and length of ownership may also impact its role in the owner’s life: puppies might be more child-like, while adult dogs could be more friend-like, though there are also differences among adult dogs^36,42^. Accordingly, we assess the effects of having children, owner age, and dog age on the relationship characteristics of the dog and, based on the results discussed above, propose the following hypotheses (for the interpretation of the scale names, see Table 1):

- Owners with child (regardless of the child’s age) will have less positive ratings of their relationship with their dogs, specifically lower ratings for overall Support, Companionship, Your Affection (i.e., how much the participant likes the partner), and Nurturance scales, compared to owners without child.
- Older and younger owners will have higher global support ratings, specifically for Companionship, Your Affection, and Nurturance, and lower negative scale ratings compared to middle-aged owners.
- Puppies exhibit a more child-like relationship pattern (i.e., higher Nurturance, Reassurance of Worth, and Your Affection, but also higher Conflict and Antagonism, and unbalanced Relative Power), while adult dogs exhibit a more friend-like relationship pattern (i.e., higher Companionship and Reliable Alliance, lower Conflict and Antagonism, and more balanced Relative Power ratings).

**Table 1:** The modified network of relationships inventory (NRI-SPV) questionnaire was used in this study.

Scale	Item	Item label
Companionship. How often do you…		
1	…and he/she go places and do things together?	Companionship1
14	…play around and have fun with him/her?	Companionship2
27	…spend fun time with him/her?	Companionship3
Instrumental Aid. How much does he/she…		
3	…teach you how to do things that you don’t know?	Instrumental Aid1
16	…help you figure out or fix things?	Instrumental Aid2
29	…help you when you need to get something done?	Instrumental Aid3
Intimacy. How often do you…		
6	…tell him/her everything that you are going through?	Intimacy1
19	…share secrets and private feelings with him/her?	Intimacy2
32	…tell him/her things that you don’t want others to know?	Intimacy3
Nurturance. How much do you…		
7	…help him/her with things she/he can’t do by her/himself?	Nurturance1
20	…protect and look out for him/her?	Nurturance2
33	…take care of him/her?	Nurturance3
Affection (for participant). How much does he/she…		
8	…like or love you?	Affection (for participant)1
21	…really care about you?	Affection (for participant)2
34	…have a strong feeling of affection (loving or liking) toward you?	Affection (for participant)3
Your Affection (towards others). How much do you…		
13	…like or love him/her?	Your affection (for others)1
26	…really care about him/her?	Your affection (for others)2
39	…have a strong feeling of affection (loving or liking) toward him/her?	Your affection (for others)3
Reassurance of Worth. How much does he/she…		
10	…treat you like you’re admired and respected?	Reassurance of Worth1
23	…treat you like you’re good at many things?	Reassurance of Worth2
36	…like or approve of the things you do?	Reassurance of Worth3
Reliable Alliance. How sure are you…		
12	…that this relationship will last no matter what?	Reliable Alliance1
25	…that your relationship will last in spite of fights?	Reliable Alliance2
38	…that your relationship will continue in the years to come?	Reliable Alliance3
Conflict. How often do you and he/she…		
2	…get mad at or get in fights with each other?	Conflict1
15	…disagree and quarrel with each other?	Conflict2
28	…argue with each other?	Conflict3
Antagonism (for participant)		
5	How much does he/she get on your nerves?	Antagonism (for participant)3
18	How much do you get annoyed with his/her behavior?	Antagonism (for participant)1
31	How much does he/she hassle or nag you?	Antagonism (for participant)2
Your antagonism (towards others)		
9	How much do you get on his/her nerves?	Your antagonism (for others)1
22	How much does he/she get annoyed with your behavior?	Your antagonism (for others)2
35	How much do you hassle or nag him/her?	Your antagonism (for others)3
Satisfaction		
4	How satisfied are you with your relationship with him/her?	Satisfaction1
17	How happy are you with the way things are between you and him/her?	Satisfaction2
30	How good is your relationship with him/her?	Satisfaction3
Relative power		
11	Who tells the other what to do more often. you or he/she?	Relative power1
24	Between you and him/her. who tends to be the BOSS in this relationship?	Relative power2
37	In your relationship with him/her. who tends to take charge and decide what should be done?	Relative power3

The number in front of each question represents their order in the questionnaire.

By addressing these research questions, this study aims to provide a comprehensive understanding of the qualitative and quantitative features of the dog-human relationship, considering various influencing factors and contemporary contexts.

## Methods

### Ethics statement

The study was reviewed and approved by the United Ethical Review Committee for Research in Psychology (EPKEB) in Hungary (reference #: 2022-77). The methods were performed in accordance with the Declaration of Helsinki. Participation was voluntary and anonymous, and informed consent was obtained from all participants.

### Procedure

The questionnaire could be filled in online (in Hungarian). It was advertised on different social media platforms and webpages in two time periods: from April 2011 to February 2013, and from January 2022 to December 2023 (after all COVID restrictions had been lifted). We used the Network of Relationships Inventory - Social Provision Version (NRI-SPV)^38^ with the same modifications as in Bonas et al.^37^. The original questionnaire contained twelve scales, each scale was measured with three questions to be rated on a 1–5 or 1–4 Likert-type (mostly frequency) scale. Of these twelve, one (Punishment) scale was removed, and two modified scales (6 questions) were added, called Your Affection and Your Antagonism. These included the same questions as the original Affection and Antagonism scales, except that they asked how the subjects felt towards the partner, rather than how the partner felt towards the subject (as in the case of the original scales). We also changed the expression “this person” in the questions to the more general “he/she”, so the questions could be answered for dogs as well. All in all, the relationship characteristics section of the questionnaire contained 39 questions aimed at assessing 13 scales (see Table 1). According to the official categorization of the scales^38^, Relative Power and Satisfaction were not part of any super-scale, seven scales (+ Your Affection, added by^37^) formed the positive super-scale Support, and two scales (+ Your Antagonism, added by^37^) formed the negative super-scale Negative Interactions.

The participants were asked to rate each question towards different potential partners. In the 2011-13 questionnaire version, the partners were mother, father, (favourite) sibling, romantic partner, best friend, and (favourite) dog. In the 2022-23 version, the partners were closest kin (mother, father, sibling, or other (grandparents, niece/nephew, etc.), defined as the relative with whom the subject currently has the closest relationship), child, romantic partner, best friend, and (favourite) dog. We reduced the number of blood kin partners to the closest kin because we did not have any specific hypothesis about potential differences between them, and because we added a new partner (child), so we wanted to reduce respondent burden. In both questionnaire versions, the subjects were to rate the characteristics of a given partner only if that partner was (still) part of the subject’s social network.

Aside from the questions about the relationship characteristics, we also inquired about basic demographic information about the subject (i.e., gender, age), and about the dog (sex, age, breed). In the 2022-23 questionnaire version, we also asked about the age of the child (if any), and whether the subject had lived or currently lives together with the four human partners (closest kin, child, romantic partner, best friend). The item response rate was above 95% for all questions and partners, indicating adequate motivation from the participants. More importantly, it also suggests that participants had no difficulty interpreting the items^43^, meaning they were able to apply the originally human characteristics in the context of their relationships with their dogs.

### Subjects

Participation in the questionnaire was voluntary; the only criterion we set was that the owner currently owned at least one companion dog. The questionnaire was completed by *N* = 717 participants, with *N* = 434 in the 2011-13 period and *N* = 283 in the 2022-23 period. The majority of respondents were female (90.2%), most of them were between 25 and 45 years old (mean age (± SD): 35.42 ± 11.75 years), and they acquired their dog, on average, within 5 years prior to participation (mean age of the dog (± SD): 4.72 ± 3.64 years, among them, *N* = 88 dogs were less than 1 year old).

The actual number of subjects in the different analyses varied, partly because those who could not or chose not to fill in the questionnaire for a specific partner were not included in that portion of the analysis. Additionally, because none of the questions were obligatory, if there was a missing question out of the three that made up a scale, we did not calculate a score for that scale for that partner. Descriptive information on the valid number of subjects eligible for analyses for each relationship scale can be found in Supplementary Table S1.

### Statistical analyses

#### Sample, partners

We compared the two sampling occasions and found significant differences in 31 out of 60 comparisons, though only two had effect sizes (Cohen’s d) reaching medium levels (see Supplementary Table S2 and Sect. 3.1 of the Results for details). While these findings justified merging the samples to increase both sample size (for identifying relationship scales) and diversity (for investigating factors influencing dog relationship ratings), the small differences could still influence comparisons between dog-human and human-human relationships. Therefore, we performed these latter analyses on both the merged sample (main text) and the separate samples (supplementary information) to ensure a consistent pattern of results.

The human partners were partly different in the datasets: the 2022-23 sample included only the kin with whom the subject felt the closest, while the 2011-13 sample included the mother, father, and sibling of the subject. Since there were some differences between these kin (Supplementary Table S3), in the latter dataset, we considered only the kin with the highest overall Support score (in the case of ties, the lowest Negative Interactions score). In 58.1% of the cases, it was the mother, in 16.1% it was the father, and in 25.8% it was the sibling of the subject. In the 2022-23 sample, the closest kin were reported to be the mother in 45.6% of the cases, the father in 10.9%, the sibling in 24.2%, and other relationships in 19.3% of the cases.

#### Relationship scales

In the case of human partners, we used the scoring manual of the questionnaire as a template and calculated the scores for the 13 relationship scales by averaging the scores given to the three questions belonging to each scale. The scores of the two super-scales (Support and Negative Interaction) were calculated by averaging the scores of the scales belonging to the super-scale. In the case of Support, we allowed one missing value, as this scale was made up of eight scales; in all other cases, no missing values were allowed. We calculated the internal consistency (using Cronbach’s alpha) of each scale to assess whether they were reliable in this sample.

In the case of dogs, we also calculated the Cronbach’s alphas for the official scales. However, since the questionnaire we used was developed for assessing human relationships, we also needed to justify the use of the same relationship scales for assessing dog-human relationships. To this end, we performed exploratory factor analysis (EFA) with Oblimin rotation on the 39 relationship questions assessed for dogs.

#### Comparison between the dog-human and human-human relationships

Next, we investigated how the dog-human relationship relates to human-human relationships. We used nonparametric tests because all scales deviated significantly from the normal distribution.

Since we had different numbers of subjects for the different human partners, we used pairwise tests instead of multivariate models. To compare the assessment of dogs to different human partners on the different relationship scales, we used the Wilcoxon signed-rank test. The effect size of the differences was estimated using Cohen’s d. Considering the large number of comparisons and the relatively large sample size, we decided to consider only the differences with at least a medium effect size (|Cohen’s d| > 0.5^44^), .

To investigate which human partner the dog most resembles when considering all relationship scales together, we created a distance index (d) from the scores given to dogs (p1,2,.,13) and the scores given to that particular human partner (q1,2,.,13) on the 13 scales, using the Euclidean distance formula (Eq. (1)).

1 $$\:d\left(p,q\right)=\:\sqrt{{({p}_{1}-{q}_{1})}^{2}+{({p}_{2}-{q}_{2})}^{2}+\dots\:+{({p}_{13}-{q}_{13})}^{2}}$$

This formula gave us a single number that represents the overall distance between the two types of relationships, the lower the score, the closer the ratings are. The distance indices of the four human partners were compared to each other using the Wilcoxon signed-rank test.

Third, to investigate if owners use the additional support from their dogs to compensate for the inadequate support of their human relationships, we investigated the correlation between the dog and human relationship scales using Spearman correlation.

#### Factors affecting the dog-owner relationship

Finally, we also investigated how different factors affect the characteristics (i.e., the thirteen relationship scales and two super-scales) of the dog-human relationship. Specifically, we assessed the effect of having child(ren) (yes, no) using Mann-Whitney U tests, and the age of the owner and dog using both Spearman correlations (age as a continuous variable) and Mann-Whitney U tests (age groups). In the case of the owners, lacking a relevant biological threshold, we divided the sample into two by the median (34 years of age). In the case of dogs, we compared the ratings of dogs below and above 1 year of age.

## Results

### Samples

When comparing the two samples collected on two occasions, we found that both owners and dogs in the 2022-23 sample were somewhat older than those in the 2011-13 sample (owners: 32.1 ± 10.1 years vs. 40.6 ± 11.2 years, z = 9.721, *p* < 0.001; dogs: 4.1 ± 3.3 years vs. 5.7 ± 4.0 years, z = 5.256, *p* < 0.001). However, there was no marked difference in the relationship characteristics between the samples for either dog or human partners. Out of all comparisons (15 relationship scales × 4 partners), only two exhibited at least a medium (|Cohen’s d| > 0.5) effect size (details in Supplementary Table S2). There were a number of significant differences with small effect sizes, suggesting a trend: owners in the 2022-23 sample rated their relationships with dogs, closest kin, and best friends less positively compared to owners in the 2011-13 sample, while their ratings for romantic partners were more positive. Specifically, the 2022-23 sample showed slightly lower ratings in Affection and Your Affection for dogs, closest kin, and best friends, but somewhat higher ratings in Instrumental Aid (one of the differences with medium effect size), Intimacy, Nurturance, and Reassurance of Worth for romantic partners. Interestingly, the Reliable Alliance increased significantly from 2011 to 13 to 2022-23 for all partners, although only in the case of dogs did this difference reach a medium effect size. Due to these mostly negligible differences, it was feasible to merge the samples for most subsequent analyses.

### Relationship scales

When we analysed the reliability of the scales for human relationships, all scales and both super-scales showed at least a good level of internal consistency (Cronbach’s alpha > 0.700) (Table 2).

**Table 2:** Internal consistency of the thirteen relationship scales and two super-sales.

	Cronbach’s alpha	
	Human relationships (combined)	Dog relationship
Support	0.961	0.889
Companionship	0.782	0.610
Instrumental Aid	0.798	0.723
Intimacy	0.917	0.895
Nurturance	0.857	0.656
Affection (for participant)	0.885	0.719
Your affection (for others)	0.894	0.788
Reassurance of worth	0.785	0.592
Reliable alliance	0.928	0.802
Negative interaction	0.921	0.849
Conflict	0.859	0.745
Antagonism (for participant)	0.817	0.718
Your antagonism (for others)	0.801	0.654
Other		
Satisfaction	0.934	0.829
Relative power	0.824	0.816

The dog-human relationship questions were subjected to an EFA to investigate if a similar scale structure would emerge as in the case of humans. We extracted 13 factors to mimic the human scale structure and found a good match to the human structure. Twelve out of the thirteen scales formed their own factor (Table 3), and only one item out of the 39 did not reach a loading > 0.3 on any factor (the minimum loading threshold in EFA^45^), , while only two items loaded on multiple factors with loadings > 0.3. There were only two discrepancies from the human structure worth mentioning: namely, the questions of the Reassurance of Worth scale loaded together with the questions of the Instrumental Aid scale, and one question seceded from both the Antagonism and Your Antagonism scales, forming a separate factor. However, it needs to be noted that only the first 9 factors had Eigenvalues > 1, while the factors corresponding to the Antagonism, Your Antagonism, and Companionship scales did not reach this threshold. Nevertheless, the scales’ reliability was also confirmed by the internal consistency assessment (Table 2), as we found an acceptable Cronbach’s alpha (> 0.6) for all scales except for the Reassurance of Worth, where the reliability was somewhat lower (Cronbach’s alpha = 0.592).

**Table 3:** Results of the EFA on the dog-human relationship questions.

	Factors												
Item labels	1	2	3	4	5	6	7	8	9	10	11	12	13
Satisfaction1	**0.630**	− 0.038	0.046	0.058	0.035	0.001	0.051	− 0.041	− 0.130	0.020	0.055	0.043	− 0.064
Satisfaction2	**0.681**	− 0.006	0.092	0.005	0.029	− 0.066	0.063	− 0.035	− 0.011	0.025	− 0.033	0.033	0.023
Satisfaction3	**0.642**	− 0.026	0.020	0.031	0.069	− 0.092	− 0.027	0.020	− 0.101	0.020	0.004	0.149	− 0.034
Conflict1	− 0.215	**0.311**	0.018	0.050	0.001	0.023	− 0.053	0.049	0.000	− 0.147	− 0.055	− 0.099	− 0.056
Conflict2	0.082	**0.747**	− 0.051	0.030	0.059	0.058	0.042	0.015	0.061	− 0.090	− 0.023	− 0.030	0.074
Conflict3	− 0.054	**0.632**	0.022	− 0.051	0.007	− 0.012	− 0.017	− 0.021	− 0.046	− 0.074	− 0.092	− 0.044	− 0.021
Reliable Alliance1	0.267	0.040	**0.310**	0.001	− 0.152	− 0.076	0.015	0.070	− 0.153	0.058	0.114	− 0.068	− 0.152
Reliable Alliance2	0.121	0.014	**0.816**	0.022	0.087	− 0.032	− 0.038	0.046	0.031	0.025	− 0.002	− 0.018	0.066
Reliable Alliance3	− 0.023	− 0.060	**0.898**	0.012	0.097	0.001	− 0.019	− 0.012	0.065	0.004	0.005	− 0.028	− 0.040
Relative Power1	0.041	0.057	− 0.021	**0.760**	− 0.051	0.002	− 0.046	0.022	0.063	0.075	− 0.086	− 0.016	0.032
Relative Power2	0.013	− 0.072	0.036	**0.703**	− 0.007	− 0.019	− 0.034	0.031	− 0.035	− 0.094	0.091	0.028	0.003
Relative Power3	− 0.046	0.019	0.000	**0.890**	0.024	0.007	0.072	− 0.096	− 0.018	0.044	0.034	− 0.022	− 0.054
Nurturance1	− 0.038	0.006	0.051	0.037	**0.355**	− 0.042	0.153	0.051	− 0.064	0.016	− 0.059	0.035	− 0.044
Nurturance2	0.028	0.042	0.080	− 0.006	**0.581**	− 0.085	− 0.005	0.008	− 0.075	0.016	0.019	− 0.020	− 0.138
Nurturance3	0.012	0.093	0.112	0.038	**0.702**	− 0.077	− 0.011	− 0.009	− 0.071	0.098	0.009	− 0.038	− 0.050
Your Affection (for others)1	0.016	− 0.046	0.015	0.022	− 0.020	**− 0.753**	0.070	− 0.047	− 0.048	− 0.030	0.025	− 0.003	− 0.001
Your Affection (for others)2	0.059	0.065	− 0.152	0.026	0.241	**− 0.428**	− 0.027	0.088	− 0.016	0.077	0.017	− 0.068	− 0.182
Your Affection (for others)3	− 0.006	− 0.021	0.050	− 0.019	0.005	**− 0.955**	− 0.012	0.016	− 0.001	− 0.040	− 0.025	0.036	0.096
Intimacy1	0.025	− 0.015	− 0.007	0.032	− 0.022	− 0.023	**0.846**	− 0.025	0.018	− 0.008	− 0.014	− 0.007	− 0.004
Intimacy2	− 0.039	0.036	− 0.008	− 0.044	0.022	− 0.018	**0.796**	0.051	− 0.065	0.013	− 0.027	0.077	− 0.079
Intimacy3	0.037	0.010	− 0.038	− 0.032	0.031	− 0.007	**0.847**	0.065	0.014	− 0.006	0.057	− 0.066	0.053
Instrumental Aid1	0.037	− 0.048	− 0.004	0.026	0.109	− 0.009	− 0.016	**0.430**	0.044	0.024	0.048	− 0.090	− 0.239
Instrumental Aid2	0.046	− 0.004	− 0.017	− 0.111	− 0.029	− 0.095	0.206	**0.642**	0.060	0.081	− 0.008	− 0.001	− 0.015
Instrumental Aid3	− 0.047	0.061	0.024	− 0.072	0.005	− 0.046	0.176	**0.665**	− 0.002	0.042	0.057	0.068	− 0.010
Reassurance of Worth1	0.223	− 0.166	0.005	0.056	0.250	0.081	0.044	0.216	− 0.294	− 0.139	− 0.032	0.027	0.209
Reassurance of Worth2	− 0.069	− 0.039	0.092	0.056	0.012	− 0.030	0.097	**0.391**	− 0.083	− 0.080	− 0.079	0.086	0.083
Reassurance of Worth3	0.129	− 0.105	− 0.020	0.124	0.107	0.046	0.010	**0.328**	− 0.189	− 0.031	0.025	0.022	0.031
Affection (for participant)1	0.038	− 0.027	0.011	0.027	0.017	− 0.061	0.061	− 0.061	**− 0.740**	0.004	0.023	− 0.014	− 0.004
Affection (for participant)2	0.067	0.041	− 0.037	− 0.030	− 0.068	− 0.127	− 0.013	**0.331**	**− 0.363**	0.022	− 0.003	0.200	− 0.069
Affection (for participant)3	0.065	− 0.018	− 0.059	− 0.007	0.046	− 0.050	0.001	− 0.016	**− 0.827**	0.032	0.035	− 0.068	− 0.005
Your Antagonism (for others)1	0.026	0.046	− 0.042	0.012	0.049	− 0.021	− 0.050	0.016	0.053	0.017	**− 0.826**	− 0.047	0.001
Your Antagonism (for others)2	0.023	0.040	0.044	− 0.080	− 0.132	0.044	0.067	− 0.026	− 0.025	− 0.126	**− 0.491**	− 0.103	− 0.085
Your Antagonism (for others)3	0.008	0.070	− 0.035	− 0.006	− 0.074	− 0.045	− 0.001	− 0.014	0.062	**− 0.593**	− 0.012	− 0.047	− 0.013
Antagonism (for participant)3	− 0.027	0.075	0.011	− 0.044	0.043	− 0.003	0.003	− 0.028	− 0.033	**− 0.714**	− 0.044	− 0.018	− 0.049
Antagonism (for participant)1	− 0.093	0.019	0.094	0.010	0.039	0.024	0.022	− 0.027	− 0.086	− 0.064	− 0.156	**− 0.686**	0.065
Antagonism (for participant)2	− 0.069	0.234	− 0.046	0.028	− 0.033	0.004	− 0.039	0.031	− 0.023	− 0.136	− 0.093	**− 0.551**	0.002
Companionship1	− 0.014	0.013	0.239	0.025	0.199	0.002	0.022	− 0.020	− 0.099	− 0.030	− 0.087	0.088	**− 0.469**
Companionship2	0.164	− 0.107	− 0.128	0.015	0.086	− 0.090	0.125	0.139	0.037	− 0.110	− 0.007	− 0.021	**− 0.504**
Companionship3	0.172	− 0.084	0.112	− 0.070	**0.540**	− 0.047	0.063	0.025	0.053	− 0.049	0.078	0.010	0.016
Initial Eigenvalues	*9.071*	*4.158*	*3.224*	*2.041*	*1.633*	*1.559*	*1.125*	*1.058*	*1.000*	*0.889*	*0.831*	*0.804*	*0.789*
Initial % of variance	*23.259*	*10.661*	*8.266*	*5.232*	*4.186*	*3.998*	*2.886*	*2.714*	*2.562*	*2.281*	*2.131*	*2.061*	*2.024*

The item labels refer to which theoretically expected relationship scale a given question belongs to (see Table 1). Loadings > 0.3 are in bold.

Next, we ran a secondary EFA (also with Oblimin rotation) on the thirteen relationship scales of the dogs to investigate the existence of the two human super-scales, Support, and Negative Interaction. The results (Table 4) showed that the eight scales that were supposed to form the Support super-scale indeed loaded together, just as the three negative relationship scales formed the Negative Interaction super-scale. The remaining two scales (Satisfaction and Relative Power) were not supposed to be categorized as either positive or negative. Our results fit this assumption, as the Satisfaction scale loaded both on Support and Negative Interaction, while Relative Power did not load on any of the super-scales. The internal consistency of the super-scales was also good (Cronbach’s alpha > 0.8, Table 2), confirming the reliability of these scales for dog-human relationships as well.

**Table 4:** Results of the secondary EFA on the dog-human relationship scales.

	Support	Negative interaction
Companionship	**0.724**	0.069
Nurturance	**0.679**	0.095
Instrumental aid	**0.638**	0.018
Affection	**0.635**	− 0.134
Your affection	**0.631**	0.000
Reassurance of worth	**0.605**	− 0.071
Intimacy	**0.591**	0.113
Satisfaction	**0.570**	− **0.342**
Reliable alliance	**0.405**	− 0.073
Antagonism	0.071	**0.831**
Your antagonism	0.069	**0.766**
Conflict	− 0.024	**0.752**
Relative power	0.038	− 0.128
Initial Eigenvalues	4.374	33.649
Initial % of variance	2.099	16.147

Loadings > 0.3 are in bold.

All in all, we found a very good correspondence between the human and dog relationship structure, justifying their use in subsequent analyses.

### Comparison between the dog-human and human-human relationships

Next, we investigated how the dog-human relationship relates to different human-human relationships.

**Fig. 1:**
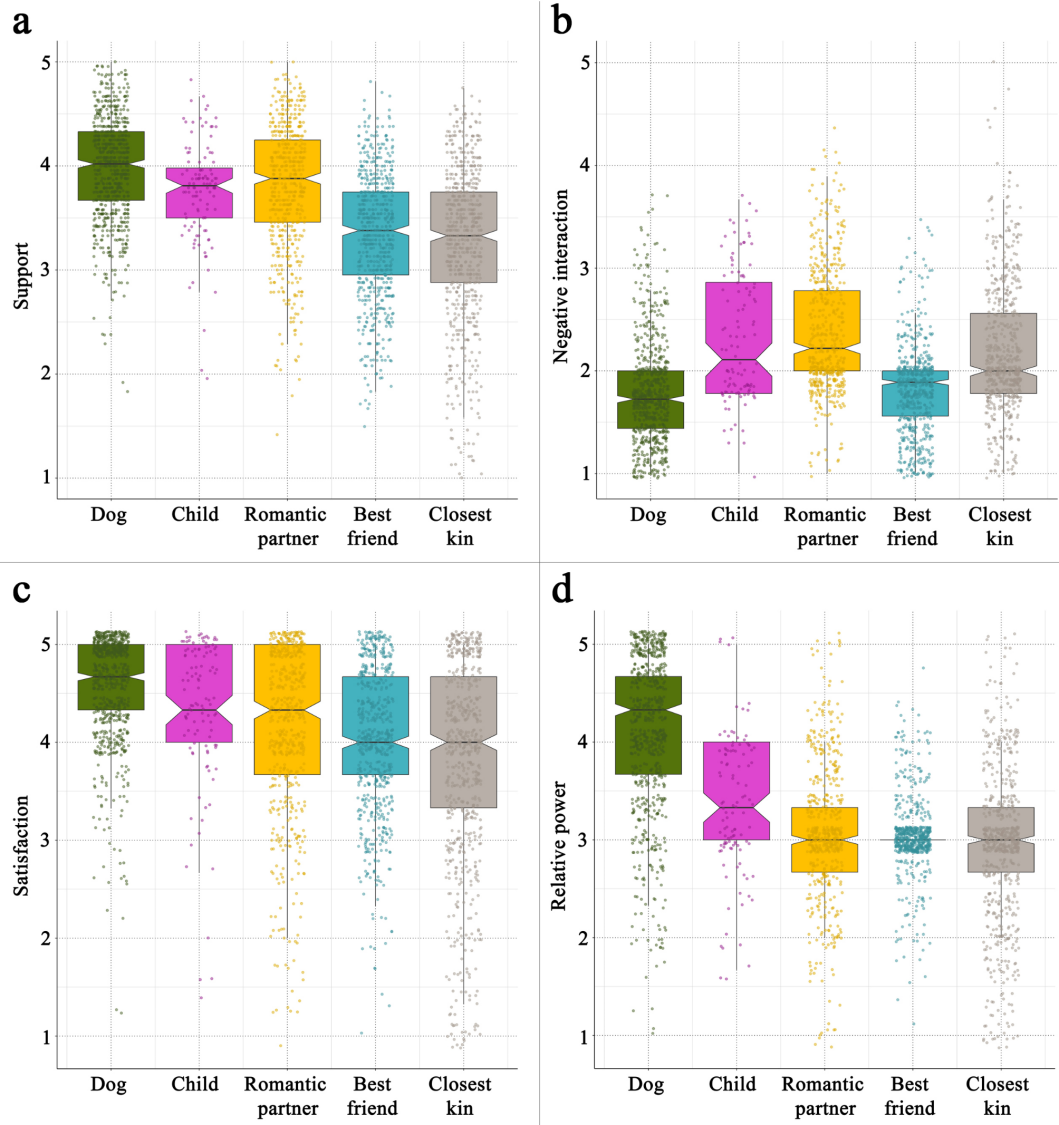
Comparison between the dog-human and human-human relationships on different relationship scales. The dogs received significantly higher ratings on overall Support (**a**) compared to all human partners except child, however, only in the cases of closest kin and best friend did not difference reach medium or higher effect size. In Negative Interaction (**b**), the dog received a lower rating than all human partners, but in the case of best friend, the effect size of the difference is negligible. The owners reported a higher rate of relationship Satisfaction (**c**) in dogs than in all human partners, but in the case of romantic partner and child, the difference had lower than medium effect size. Regarding Relative Power (**d**), the dog received a markedly higher rating than all human partners.

First, we compared the ratings of each relationship characteristic between the dog and the four human partners. The results of the merged sample can be found in Table 5. Out of the 60 comparisons, only four did not reach statistical significance; however, only 34 differences had at least a medium effect size (|Cohen’s d| > 0.5). Accordingly, the dogs received higher ratings for the scales Companionship and Nurturance than closest kin, best friend, and romantic partner. The owners also rated dogs higher on the super-scale Support (Fig. 1a), and on the scales Affection, Your Affection, Reassurance of Worth, and Reliable Alliance, compared to closest kin and best friend. However, we found only a below-medium difference in Instrumental Aid between the dog and the human partners, while in Intimacy, the romantic partner received a higher score than the dog. We found no difference with at least medium effect size between the relationship ratings of dogs and children in any of the positive relationship scales.

**Table 5:** Results of the pairwise comparisons between the ratings of the dog and of the four human partners (Wilcoxon signed rank test).

Relationship scales	Closest kin				Child				Romantic partner				Best friend			
	*N*	z value	*p* value	Cohen’s d (95% CI)	*N*	z value	*p* value	Cohen’s d (95% CI)	*N*	z value	*p* value	Cohen’s d (95% CI)	*N*	z value	*p* value	Cohen’s d (95% CI)
Support	**640**	− **17.906**	*p* < 0.001	− **0.866 (**− **0.957 to 0.775)**	109	− 0.198	0.843	− 0.035 (− 0.223 to 0.153)	529	− 4.236	*p* < 0.001	− 0.226 (− 0.312 to 0.140)	**645**	− **18.559**	*p* < 0.001	− **0.959 (**− **1.052 to 0.866)**
Companionship	**653**	− **21.641**	*p* < 0.001	− **1.812 (**− **1.937 to 1.687)**	111	− 3.842	*p* < 0.001	− 0.387 (− 0.579 to 0.194)	**541**	− **14.065**	*p* < 0.001	− **0.735 (**− **0.830 to 0.640)**	**652**	− **20.878**	*p* < 0.001	− **1.514 (**− **1.626 to 1.401)**
Instrumental aid	643	− 1.876	0.061	− 0.101 (− 0.178 to 0.023)	109	− 3.615	*p* < 0.001	0.356 (0.161 to 0.548)	535	− 10.025	*p* < 0.001	0.469 (0.380 to 0.558)	647	− 2.781	0.005	− 0.120 (− 0.197 to 0.042)
Intimacy	646	− 4.916	*p* < 0.001	− 0.204 (− 0.281 to 0.126)	108	− 1.927	0.054	0.175 (− 0.015 to 0.365)	**537**	− **11.422**	*p* < 0.001	**0.558 (0.467 to 0.648)**	654	− 7.300	*p* < 0.001	0.309 (0.230 to 0.387)
Nurturance	**648**	− **19.127**	*p* < 0.001	− **1.088 (**− **1.185 to 0.990)**	109	− 1.252	0.211	− 0.106 (− 0.294 to 0.082)	**530**	− **11.249**	*p* < 0.001	− **0.548 (**− **0.639 to 0.457)**	**649**	− **19.993**	*p* < 0.001	− **1.250 (**− **1.352 to 1.147)**
Affection (for participant)	**649**	− **13.655**	*p* < 0.001	− **0.605 (**− **0.688 to 0.521)**	112	− 2.891	0.004	− 0.290 (− 0.479 to 0.101)	540	− 4.875	*p* < 0.001	− 0.211 (− 0.297 to 0.126)	**650**	− **18.984**	*p* < 0.001	− **1.098 (**− **1.195 to 1.001)**
Your affection (for others)	**658**	− **16.125**	*p* < 0.001	− **0.733 (**− **0.819 to 0.647)**	112	− 4.844	*p* < 0.001	0.457 (0.261 to 0.650)	541	− 6.487	*p* < 0.001	− 0.302 (− 0.388 to 0.216)	**650**	− **19.734**	*p* < 0.001	− **1.207 (**− **1.308 to 1.106)**
Reassurance of worth	**632**	− **15.248**	*p* < 0.001	− **0.747 (**− **0.835 to 0.659)**	109	− 2.170	0.030	− 0.202 (− 0.391 to 0.012)	525	− 8.232	*p* < 0.001	− 0.388 (− 0.477 to 0.300)	**632**	− **13.634**	*p* < 0.001	− **0.620 (**− **0.705 to 0.535)**
Reliable alliance	651	− 11.095	*p* < 0.001	− 0.455 (− 0.536 to 0.375)	110	− 2.946	0.003	− 0.281 (− 0.471 to 0.090)	**542**	− **15.063**	*p* < 0.001	− **0.808 (**− **0.905 to 0.711)**	**656**	− **16.986**	*p* < 0.001	− **0.843 (**− **0.932 to 0.754)**
Negative interaction	**612**	− **13.395**	*p* < 0.001	**0.592 (0.506 to 0.677)**	**105**	− **7.268**	*p* < 0.001	**0.893 (0.665 to 1.118)**	**504**	− **15.572**	*p* < 0.001	**0.877 (0.774 to 0.980)**	612	− 2.054	0.040	0.044 (− 0.035 to 0.123)
Conflict	**644**	− **14.017**	*p* < 0.001	**0.631 (0.546 to 0.715)**	**107**	− **7.174**	*p* < 0.001	**0.964 (0.733 to 1.192)**	**534**	− **14.576**	*p* < 0.001	**0.772 (0.675 to 0.868)**	641	− 4.096	*p* < 0.001	0.132 (0.054 to 0.210)
Antagonism (for participant)	642	− 7.900	*p* < 0.001	0.331 (0.252 to 0.411)	**108**	− **4.797**	*p* < 0.001	**0.520 (0.318 to 0.720)**	**536**	− **10.967**	*p* < 0.001	**0.534 (0.444 to 0.625)**	652	− 5.050	*p* < 0.001	− 0.199 (− 0.276 to 0.121)
Your antagonism (for others)	**649**	− **14.740**	*p* < 0.001	**0.666 (0.581 to 0.751)**	**112**	− **7.587**	*p* < 0.001	**0.945 (0.720 to 1.166)**	**536**	− **17.304**	*p* < 0.001	**1.099 (0.992 to 1.206)**	653	− 7.089	*p* < 0.001	0.274 (0.196 to 0.352)
Satisfaction	**650**	− **15.409**	*p* < 0.001	− **0.709 (**− **0.795 to 0.623)**	110	− 3.241	0.001	− 0.318 (− 0.509 to 0.126)	544	− 10.234	*p* < 0.001	− 0.481 (− 0.570 to 0.392)	**646**	− **14.710**	*p* < 0.001	− **0.696 (**− **0.781 to 0.609)**
Relative power	**640**	− **19.115**	*p* < 0.001	− **1.131 (**− **1.230 to 1.032)**	**110**	− **7.227**	*p* < 0.001	− **0.914 (**− **1.135 to 0.690)**	**532**	− **16.793**	*p* < 0.001	− **1.025 (**− **1.130 to 0.920)**	**643**	− **19.346**	*p* < 0.001	− **1.246 (**− **1.349 to 1.143)**

The effect size of the difference and its 95% confidence interval is also provided. Negative values indicate that the dog received higher values than the human partner. Differences with at least medium effect size (|Cohen’s d| > 5) are in bold.

Regarding the negative scales, the dog received a lower rating than the closest kin, child, and romantic partner in the Negative Interaction super-scale (Fig. 1b), as well as in the Conflict and Your Antagonism scales, and a lower rating than the child and romantic partner in the Antagonism scale. We found no difference with at least medium effect size between dogs and best friends in any of the negative relationship scales. In the remaining two scales, the owners reported a higher rate of relationship Satisfaction in dogs than in closest kin and best friend (but not in child and romantic partner, Fig. 1c). Regarding Relative Power, the dog received a higher rating than all human partners (Fig. 1d), indicating that the power balance is more shifted in the relationship with dogs than with any human partner.

Since merging the two datasets might still bias the results, despite the small effect size of the differences, we conducted these comparisons both on the merged sample (main text) and separately on the 2011–2013 and 2022–2023 samples (see supplementary information).

Regarding the 2022–2023 sample (Supplementary Table S4), we found a high level of correspondence (91.1%) with the merged sample. Since the dog-child comparison was conducted only on the 2022–2023 sample, it was excluded from the correspondence analysis. Of the remaining 29 differences with medium effect sizes in the merged sample, 27 were consistent in this sample as well, both in direction and effect size. The two differences that did not reach medium effect size in this sample were Your Antagonism and Negative Interaction of the closest relative (Cohen’s d = 0.457 and 0.462, respectively). Of the 16 small or non-significant differences in the merged sample, 14 were also consistent in this sample, both in direction and effect size. Some of these differences did not even reach statistical significance, likely due to the lower sample size. The two differences that reached medium effect size in this sample were Reliable Alliance of the closest relative (Cohen’s d = -0.541) and Instrumental Aid of the romantic partner (Cohen’s d = 0.755).

Regarding the 2011–2013 sample (Supplementary Table S5), the correspondence with the merged sample was 95.5%. Of the differences with at least medium effect size in the merged sample, only one did not reach medium effect size in this sample: Intimacy of the romantic partner (Cohen’s d = 0.409). Similarly, of the 16 small or non-significant differences in the merged sample, only one reached medium effect size in this sample: Satisfaction of the romantic partner (Cohen’s d = -0.519).

All in all, the two samples separately showed a very similar pattern of differences, particularly regarding differences with at least medium effect sizes. This supports our decision to merge the samples and also to focus on effect size rather than statistical significance.

**Fig. 2:**
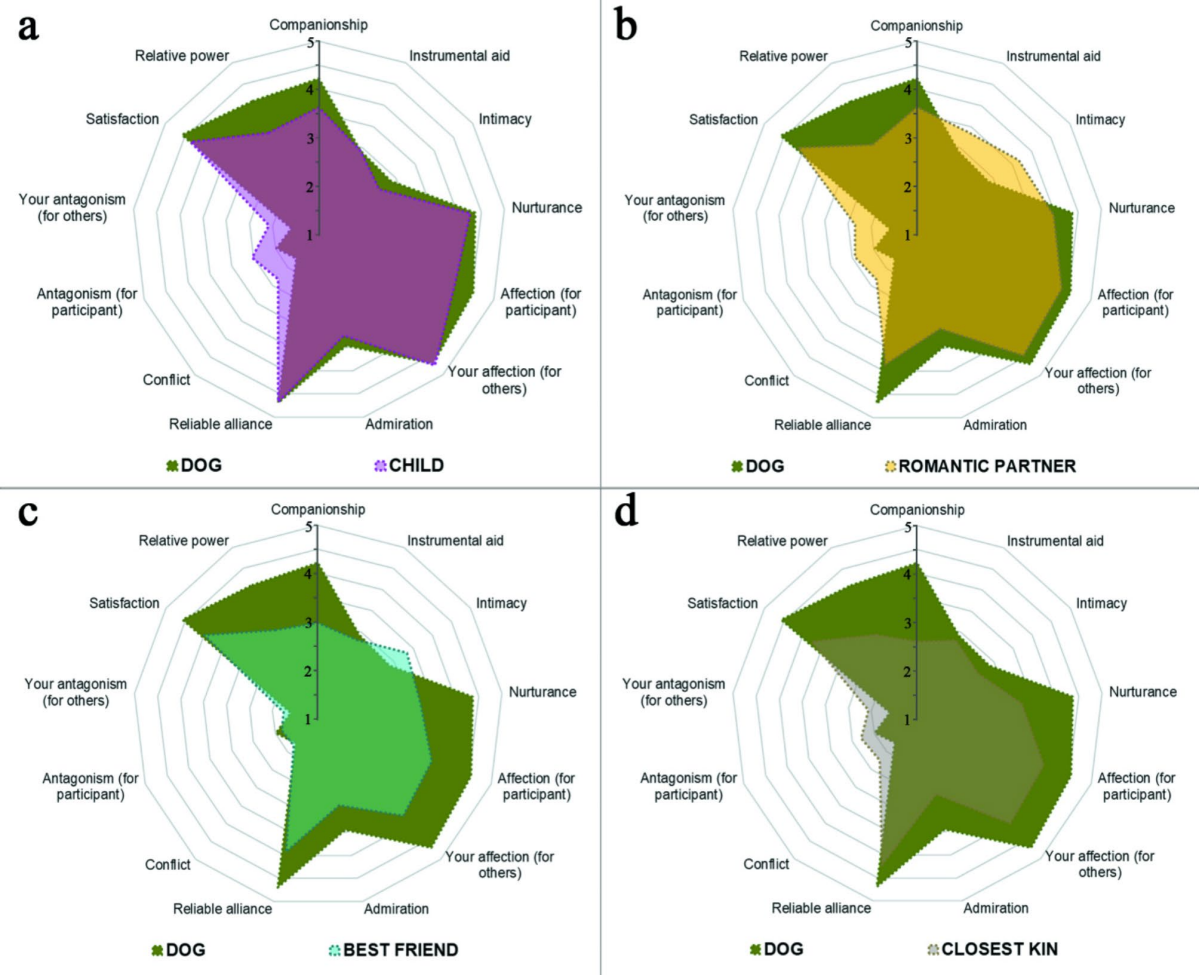
Sematic representation of the overlap between the dog-human and different human-human relationship ratings. The panels represent the relationship profile of the dog and child (**a**), dog and romantic partner (**b**), dog and best friend (**c**), and dog and closest kin (**d**). Each of the 13 axes on the spiderweb represents a distinct relationship quality. For each quality, the distance from the center represents the average rating of that relationship quality for a given partner. Concentric lines represent a 0.5 change in the value.

Second, to investigate the similarity between the dog-human and human-human relationships, we created a distance index from different relationship scales based on the Euclidean distance formula. We found that, globally, the child-parent relationship was the closest to the human-dog relationship (*p* < 0.001, |Cohen’s d| > 0.5 for all paired comparisons, Table 6). However, even this relationship did not fully reflect the pattern of the dog-human relationship (Fig. 2), suggesting that the dog-owner relationship has a unique pattern. Regarding the order of the other relationships, romantic partners were more similar to the dog than best friends and closest kin (*p* < 0.001), and best friends were more similar than closest kin (*p* = 0.005). However, the effect size of these latter comparisons was small (|Cohen’s d| < 0.5). The same pattern of results could be observed if the distance index was calculated only from the positive scales, while no difference of at least medium effect size could be observed if the distance index was calculated only from the negative scales.

**Table 6:** Results of the pairwise comparisons of the distance to the dog index between the four human partners (Wilcoxon signed rank test).

Pairs	Global (all scales)				Support				Negative interaction			
	*N*	z value	*p* value	Cohen’s d (95% CI)	*N*	z value	*p* value	Cohen’s d (95% CI)	*N*	z value	*p* value	Cohen’s d (95% CI)
Child vs. romantic partner	**93**	− **5.412**	**< 0.001**	− **0.612 (**− **0.832 to 0.389)**	**98**	− **5.640**	**< 0.001**	− **0.593 (**− **0.806 to 0.377)**	94	− 2.434	0.015	− 0.230 (− 0.434 to 0.025)
Child vs. best friend	**86**	− **4.851**	**< 0.001**	− **0.592 (**− **0.819 to 0.361)**	**90**	− **6.020**	**< 0.001**	− **0.792 (**− **1.027 to 0.553)**	86	− 3.116	0.002	0.357 (0.138 to 0.574)
Child vs. closest kin	**86**	− **6.143**	**< 0.001**	− **0.744 (0.503 to 0.981)**	**90**	− **6.754**	**< 0.001**	− **0.803 (**− **0.564 to 1.040)**	88	− 1.596	0.110	0.214 (0.425 − 0.002)
Romantic partner vs. best friend	460	− 3.921	< 0.001	− 0.164 (− 0.256 to 0.072)	502	− 7.194	< 0.001	− 0.318 (− 0.407 to 0.228)	472	− 9.332	< 0.001	0.457 (0.362 to 0.551)
Romantic partner vs. closest kin	457	− 5.457	< 0.001	− 0.285 (− 0.192 to 0.379)	500	− 6.852	< 0.001	− 0.350 (− 0.260 to 0.440)	469	− 4.237	< 0.001	0.181 (0.272 − 0.090)
Best friend vs. closest kin	572	− 2.807	0.005	− 0.198 (− 0.116 to 0.281)	623	− 0.979	0.328	− 0.139 (− 0.060 to 0.217)	586	− 6.405	< 0.001	− 0.284 (− 0.202 to 0.367)

The effect size of the difference and its 95% confidence interval is also provided. Negative values indicate that the first member of the pair has a smaller distance index value (is more similar to the dog) than the second member. Distance indexes were calculated from all scales (global), only positive scales (Support), and only negative scales (Negative Interaction). Differences with at least medium effect size (|Cohen’s d| > 5) are in bold.

We repeated this analysis on both subsamples, and in both cases, we found largely the same pattern as in the merged sample (Supplementary Table S6). Not counting the comparisons between child and other human relationships (as these were conducted only on the 2022-23 sample), we found no differences that reached medium effect size in the 2022–2023 sample – just as in the merged sample. Regarding the 2011–2013 sample, there was only one difference with a medium effect size: the similarity to friends on negative scales was higher than the similarity to romantic partners. Overall, this analysis further confirms that the two subsamples can be merged and that the pattern of results is robust.

Finally, we also investigated the correlation between the dog and human relationship scales and found either no or positive association (ρ = 0.080–0.505). Regarding the partners, on average, the child rating had the strongest (positive) correlation with the dog rating (mean = 0.311, range: 0.096–0.482), while in the case of the relationship scales, we found the strongest (positive) correlation, on average, between the dog and human partners in Reliable Alliance (mean ρ = 0.379) and Nurturance (mean ρ = 0.370).

### Factors affecting the dog-owner relationship

Among the potential factors influencing the dog-owner relationship, we investigated the effect of having a child, the age of the owner, and the age of the dog.

Regarding parenthood, owners without children rated their dogs higher both on the Support and Negative Interaction super scales, as well as specifically on the Companionship, Instrumental Aid, Intimacy, Nurturance, Affection, and Your Affection scales from the positive scales, and on the Your Antagonism scale from the negative scales. However, among these, only the differences in Companionship and Your Affection had at least a medium effect size (|Cohen’s d| > 0.5) (Table 7).

**Table 7:** Results of the association of the dog relationship ratings with parenthood, and dog and owner age.

Relationship scales	Having child or not				Correlation with owner age		Correlation with dog age	
	*N*	z value	*p* value	Cohen’s d (95% CI)	Spearman’s ρ	*p* value	Spearman’s ρ	*p* value
Support	270	− 3.854	< 0.001	0.441 (0.194 to 0.686)	− 0.087	0.023	− 0.044	0.249
Companionship	**282**	− **4.185**	**< 0.001**	**0.513 (0.271 to 0.754)**	− 0.054	0.149	− 0.138	< 0.001
Instrumental aid	273	− 2.201	0.028	0.258 (0.015 to 0.501)	− 0.099	0.009	− 0.085	0.025
Intimacy	274	− 2.915	0.004	0.392 (0.147 to 0.635)	− 0.194	< 0.001	− 0.002	0.953
Nurturance	275	− 2.493	0.013	0.324 (0.081 to 0.566)	0.097	0.011	0.030	0.429
Affection (for participant)	280	− 2.554	0.011	0.280 (0.040 to 0.520)	− 0.081	0.032	0.007	0.853
Your affection (for others)	**279**	− **5.118**	**< 0.001**	**0.576 (0.332 to 0.820)**	− 0.157	< 0.001	− 0.008	0.826
Reassurance of worth	264	− 0.293	0.769	0.008 (− 0.237 to 0.253)	0.069	0.074	− 0.035	0.363
Reliable alliance	273	− 0.104	0.917	0.017 (− 0.225 to 0.258)	0.246	< 0.001	0.105	0.006
Negative interaction	267	− 2.537	0.011	0.312 (0.066 to 0.558)	− 0.031	0.427	− 0.026	0.499
Conflict	269	− 1.684	0.092	0.227 (− 0.017 to 0.471)	0.000	0.993	− 0.057	0.135
Antagonism (for participant)	276	− 1.355	0.175	0.180 (− 0.061 to 0.421)	− 0.031	0.407	− 0.059	0.119
Your antagonism (for others)	281	− 3.241	0.001	0.433 (0.192 to 0.673)	− 0.062	0.103	0.008	0.841
Satisfaction	275	− 0.477	0.633	− 0.029 (− 0.270 to 0.212)	0.042	0.272	0.075	0.048
Relative power	274	− 0.757	0.449	− 0.076 (− 0.317 to 0.165)	0.037	0.323	− 0.087	0.022

In the former comparison, the effect size of the difference and its 95% confidence interval is also provided. Positive values indicate that non-parents rated that scale higher than parents. Differences with at least medium effect size (|Cohen’s d| > 5) are in bold.

We found twelve significant correlations between the dog relationship ratings and the owner age and dog age. All of these correlations were negligible in strength (ρ = -0.194–0.246 for owner age and ρ = -0.138–0.105 for dog age, Table 7). Finally, regarding the age groups, we found several significant differences between owners below and above 34 years, which overlapped well with differences we found based on parenthood status. Younger owners rated their dogs higher on the Support super scale, specifically on the Companionship, Instrumental Aid, Intimacy, Affection, and Your Affection scales. They also rated their dogs higher on the Antagonism scale, but lower on the Reliable Alliance scale. None of these differences reached at least a medium effect size (|Cohen’s d| > 0.5) (Table 8). In the case of the puppy-adult dog comparison, puppies were rated higher on Companionship, lower on Reliable Alliance, and also showed a stronger shift on Relative Power towards the owner (z = 2.364; *p* = 0.018; Cohen’s d = 0.236). Again, none of these differences had at least medium effect size (Table 8).

**Table 8:** Results of the comparisons between the age groups of the owner (below vs. above 34 years of age) and the dog (below vs. above 1 year of age) on the relationship ratings (Mann–Whitney U test).

Relationship scales	Owner age < 34 year. vs. > 34 year.				Dog age < 1 year. vs. > 1 year.			
	*N*	z value	*p* value	Cohen’s d (95% CI)	*N*	z value	*p* value	Cohen’s d (95% CI)
Support	687	− 2.421	0.015	0.132 (− 0.018 to 0.281)	685	− 0.403	0.687	0.0110 (− 0.215 to 0.237)
Companionship	710	− 1.977	0.048	0.146 (− 0.001 to 0.293)	707	− 2.448	0.014	0.227 (0.004 to 0.451)
Instrumental aid	691	− 2.343	0.019	0.165 (0.016 to 0.314)	688	− 0.888	0.375	0.093 (− 0.133 to 0.319)
Intimacy	695	− 4.803	< 0.001	0.369 (0.219 to 0.519)	693	− 0.004	0.997	0.002 (− 0.225 to 0.229)
Nurturance	696	− 1.592	0.111	− 0.102 (− 0.25 to 0.047)	694	− 0.797	0.426	− 0.096 (− 0.323 to 0.131)
Affection (for participant)	699	− 2.108	0.035	0.121 (− 0.028 to 0.269)	697	− 0.499	0.618	0.065 (− 0.163 to 0.293)
Your affection (for others)	704	− 3.559	< 0.001	0.217 (0.069 to 0.365)	702	− 0.115	0.908	− 0.060 (− 0.285 to 0.164)
Reassurance of worth	675	− 1.584	0.113	− 0.137 (− 0.288 to 0.014)	673	− 0.717	0.474	− 0.075 (− 0.304 to 0.155)
Reliable alliance	699	− 5.766	< 0.001	− 0.348 (− 0.497 to 0.199)	697	− 2.233	0.026	− 0.114 (− 0.338 to 0.109)
Negative interaction	665	− 1.541	0.123	0.110 (− 0.042 to 0.262)	663	− 0.016	0.987	− 0.012 (− 0.241 to 0.217)
Conflict	691	− 0.363	0.716	0.035 (− 0.114 to 0.184)	688	− 0.816	0.414	0.044 (− 0.179 to 0.268)
Antagonism (for participant)	696	− 2.004	0.045	0.113 (− 0.036 to 0.261)	693	− 0.338	0.736	− 0.012 (− 0.240 to 0.216)
Your antagonism (for others)	700	− 1.485	0.137	0.118 (− 0.030 to 0.266)	698	− 0.136	0.892	− 0.041 (− 0.266 to 0.184)
Satisfaction	701	− 0.886	0.375	− 0.097 (− 0.246 to 0.051)	699	− 0.561	0.575	0.055 (− 0.170 to 0.280)
Relative power	696	− 1.295	0.195	− 0.110 (− 0.259 to 0.038)	694	− 2.364	0.018	0.236 (0.008 to 0.464)

Positive values indicate that the younger age group received higher values than the older age group. None of the differences had at least medium effect size (|Cohen’s d| > 5).

## Discussion

Despite extensive research on the human-animal bond^46–48^, there is limited research on the characteristics of the human-dog relationship, particularly in ways that are comparable to human relationships. This study offers a multidimensional description of the dog-owner relationship along characteristics akin to human social relationships. Our findings suggest that:

1. Owners rate their relationship with their dogs as good or better than any human relationships.
2. There is a weak to moderate positive correlation between dog and human relationship ratings, suggesting that relationships with dogs complement rather than compensate for relationships with human partners.
3. The dog-human bond has a unique pattern of relationship characteristics, with the closest resemblance to the child-parent relationship.
4. The characteristics of the dog-owner relationship change only minimally over time and in response to owner-related factors, such as parenthood.

Our first objective was to demonstrate that the relationship with a dog can be characterized using the same scales that describe human relationships. Using the same questionnaire as Bonas et al.^37^, which investigated two higher-order factors (Support and Negative Interaction), we performed an exploratory factor analysis on the dog rating items. Thirteen relationship scales emerged, with all but the Reassurance of Worth scale appearing as separate factors. The items of this latter scale loaded with those from the Instrumental Aid scale, and its internal consistency was lower, indicating a challenge in evaluating this characteristic in dog-human relationships.

The results showed that items from the Companionship, Antagonism, and Your Antagonism scales loaded on different factors, with their Eigenvalues being less than 1, suggesting lower variability. However, all scales, except for Reassurance of Worth, showed acceptable internal consistency. Furthermore, the higher-order factors corresponded well with the two super-scales in human relationships (Support and Negative Interaction), confirming the findings of Bonas et al.^37^. These results justify the use of these scales in subsequent analyses.

Our second question addressed how owners evaluate their relationship with their dogs in comparison to human relationships. We extended the research of Bonas et al.^37^ and compared dog-human relationships to various human relationships.

In terms of positive characteristics, dogs were rated at the same level as children and higher than most human partners in scales such as Companionship, Nurturance, Affection, Your Affection, Reassurance of Worth, Reliable Alliance, and the Support super-scale. Only romantic partners were rated higher than dogs, and only in the Intimacy scale. On negative scales, dogs were rated similarly to best friends but lower than all other human partners. Owners reported higher Satisfaction with dogs than with closest kin and best friends, but not higher than with children and romantic partners. Regarding Relative Power, dogs received higher ratings than all human partners, indicating a greater power imbalance toward the owner.

These results were largely in line with our hypotheses and the findings of Bonas et al.^37^. The latter study found that participants received more cumulative social support from human relationships (corresponding to close kin in our analyses) but experienced less conflict in their relationships with pets compared to human partners. While the latter finding was supported by our results, the former was not: we found higher support in dogs not only than closest kin but also than best friends. Bonas et al.^37^ also found that dogs scored higher in Companionship, Nurturance, and Reliable Alliance but lower in Instrumental Aid and Intimacy. These results were mostly confirmed by our data. However, we found that dogs performed better than most human partners not only in these three scales but in three additional scales as well, and human partners did not outperform dogs in Instrumental Aid (i.e., no meaningful difference was found), only in Intimacy. Overall, our results revealed a somewhat more positive assessment of the dog-owner relationship compared to Bonas’ study. This difference may reflect changes in dog-keeping trends over the past 20 years, although the fact that both of our samples showed the same pattern in the vast majority of cases, suggests that the key differences between dog and human partners are robust and remain relatively stable over time, even amid significant events such as the COVID-19 pandemic. Thus, discrepancies between our and Bonas’ study are (also) likely due to cultural differences.

Our findings suggest that dogs provide significant support in Companionship and Nurturance, fulfilling these social needs most effectively. Both characteristics are commonly stated reasons for dog ownership and offer intrinsic rewards. Companionship involves shared pleasure in recreation and spontaneity^49^, similar to what we typically experience with friends^13^. Nurturance, which encompasses caregiving behaviours and oxytocin system activation, reduces stress and offers social rewards^50–53^. The nurturing role of dogs may explain the growing popularity of small brachycephalic breeds, despite serious health and welfare concerns raised by veterinary professionals. The “baby schema” (i.e., infant-like facial features^54^) and dependent behaviours typical of these breeds^55^ might trigger caregiving responses from owners. In addition to these positive attributes, an important characteristic of the dog-human relationship is the lack of conflict and antagonism, at least in our sample. While human partners can both support and hinder social needs, causing stress through negative interactions, dogs have limited potential to cause distress due to the power imbalance inherent in the dog-owner relationship^40^. In (adult) human relationships, attachment bonds and relative power dynamics are typically symmetrical, but in dog-owner relationships, owners predominantly make the decisions, minimizing the likelihood of dogs sabotaging the relationship.

Aside from Bonas et al.^37^, other studies have found similar, though, not directly comparable, results to ours. For example, 62% of Hungarian dog owners in a convenience sample agreed that their dog is more important than any human^56^, although this ratio was only 12% in a representative sample^57^. Relationships with pets were shown to be more secure than those with romantic partners^33^, in line with our findings on Reliable Alliance. Owners in a Dutch convenience sample also expressed high satisfaction with their relationships with dogs^31^. Overall, at least in convenience samples, owners perceive their relationship with their dogs as superior to any human relationship. The question is, why?

One practical explanation could be that the questionnaire scales and endpoints may not represent the same concepts for dogs and human partners. Owners may interpret the scales relatively, comparing each partner to what they expect or consider ideal for each type of relationship. This could partly explain the disproportionately positive evaluation of dogs, particularly on scales like Instrumental Aid, Intimacy, and Reassurance of Worth. Future studies should aim to develop more objective scales to minimize this bias.

Another possible explanation is that dogs exceed human relationship ratings not because owners rate dogs exceptionally high, but because they rate their human relationships comparatively low. In other words, dogs may serve as substitutes for less satisfactory human relationships^58^. Research has shown that people with fewer human relationships are more likely to own companion animals^3,37^ and tend to score higher on pet attachment scales^59^. Vulnerable populations, such as the elderly, lonely individuals, and those with psychological or physical disabilities, benefit the most from pets and form stronger bonds with them^47,60,61^. However, studies by^37,40,41^ suggest that pets do not compensate for dysfunctional human relationships, but instead uniquely contribute to their owners’ well-being. Our results also support this latter conclusion. We observed null or positive correlation between dog and human relationship ratings, suggesting that dog owners do not rely on their dogs to compensate for deficiencies in their human relationships. However, it is important to note that our sample was comprised of volunteers, primarily recruited through social media, which likely led to a bias toward participants with positive attitudes toward dogs and a low representation of dissatisfied owners or vulnerable individuals^31^. In support of this, most participants reported having a romantic partner, indicating that owners without interpersonal relationships were less likely to take part in this study.

Another important consideration is that we could only compare the relationship with the dog to the closest human relationship the owner currently has (e.g., best friend, closest kin), not their full social network. The absence of a negative correlation only means that support from dogs is not perceived as better than what owners experience in their closest human relationships, but it does not address the owners’ overall need fulfilment from their entire social network. Therefore, it remains possible that the number of emotional bonds an owner has may negatively relate to the quality of their bond with the dog. Future research is needed to clarify this link.

An additional explanation for the overly positive evaluation of dogs is cognitive bias, such as pet-enhancement or social desirability bias. El-Alayli et al.^62^ found that owners tend to rate their companion animals more favourably than average pets, similar to self-enhancement biases observed in human relationships. People also have overly positive evaluations of their friends, compared to others^63^, and even develop favourable views of their possessions (i.e., mere ownership effect^64^), . This bias could explain why owners believe their dogs improve their lives, even when empirical research does not support this view^48,65^. Such biases are stronger with higher levels of pet attachment and perceived similarity^62^, and they serve to help maintain a positive self-image^64^.

Owners may also overrate their dogs to present a positive image to others. Social desirability bias refers to the tendency of individuals to conform to social norms or engage in behaviours that are seen as socially acceptable or favourable to avoid judgment or criticism. Unlike (adult) human relationships, where both partners may share responsibility for a relationship’s shortcomings, the dog-owner relationship typically places full responsibility on the owner. The notion that “There’s no such thing as a bad dog, only bad owners”, a popular sentiment in dog and pet-related social media groups, may lead owners to believe that any problem with their dog or the dyadic relationship is predominantly their fault and that they will be judged by others for it^66^.

Viewing one’s dog through rose-coloured glasses could be harmful if it prevents owners from exploring alternative ways to improve their self-worth, or if it leads them to downplay, ignore, or deny the existence of potentially dangerous behavioural problems in their dogs^62^. Additionally, it could alienate the owner from peers who hold a more realistic view of their dog.

Contrary to the previous hypotheses which assumed that owners overrate their relationship with their dogs, the fourth possible explanation is that the high ratings genuinely reflect the good quality of the dog-human bond. Some individuals may prefer dogs over other (human) attachment figures due to lower levels of avoidance and anxiety in their relationships with dogs^67^. Kurdek^11^ found that people are more likely to turn to their dogs than to most human partners (including close kin, children, and best friends, but not romantic partners) during times of emotional stress. Factors such as being male, widowed, highly involved in dog care, and uncomfortable with self-disclosure^11^, as well as having high attachment avoidance and anxiety^67^, increase the likelihood of seeking comfort from dogs instead of other humans in stressful situations. The “optimally discrepant social others” theory suggests that some individuals find it easier to interact with and open up to animals than humans. Animals are *“sufficiently similar to humans to elicit prosocial behaviuor and positive affect*,* and sufficiently dissimilar to avoid posing a threat”* (Corson and Corson, 1980, cited in^48^). Dogs are seen as loyal, non-critical, and capable of providing comfort, companionship, unconditional affection, and attention^27,68–71^. These traits enable dogs to meet owners’ needs for autonomy, competence, and relatedness^40^, which can be particularly appealing to individuals with anxious attachment styles, who often worry about being accepted or rejected by others^46^. Moreover, unlike human relationships, where dissatisfaction may lead to enduring conflict or the inability to escape, individuals dissatisfied with their dogs can opt to adopt a new one or rehome the current pet, a flexibility not available with human kin. Therefore, it is not surprising that individuals who currently keep dogs and choose to participate in a dog-related tend to express high satisfaction with their relationship, particularly if the survey focuses on their favourite dog.

We compared the similarity between dogs and human partners across all scales combined to determine whether the dog-owner relationship follows a unique pattern or aligns with any specific type of human relationship. We hypothesized that the dog-human relationship would most closely resemble the child-parent relationship and be more similar to chosen partners (such as best friends and romantic partners) than to closest kin. Our results supported the first hypothesis but only partially the second: while romantic partners were more similar to dogs than closest kin, best friends were not. In terms of negative relationship characteristics, the best friend was the most similar to the dog, although the differences were smaller compared to those observed in the positive scales. One possible explanation for the similarity in perceived relationship quality between dogs and children may lie in the relatively shorter duration of these relationships compared to those with other partners (i.e., the average length of relationships with dogs in our sample was less than five years). However, we did not collect data on the duration of other relationship types, nor the average amount of time spent with different partners, both of which could have influenced the ratings of relationship quality.

Nonetheless, our results indicate that the dog-human relationship possesses a unique pattern, distinct from the relationship profiles of any human partner.

Our third question focused on potential variations among owners and dogs regarding the role of the dog in the owner’s social network. Specifically, how do factors such as parenthood, owner age, dog age, and the timing of the survey influence the characteristics of the dog-human relationship? Based on the work of Albert & Bulcroft^3^ and Turner^14^, we hypothesized that the dog’s role would be linked to the owner’s life stage. Young adult owners may view their dog as a child-precursor, while older owners, whose children have “left the nest”, may regard the dog as a substitute, likely leading to more positive ratings of the dog-owner relationship, particularly in terms of global Support, Companionship, Nurturance, and Your Affection scales. Additionally, Blouin^27^ found that after the birth of a first child, owners’ humanistic orientation towards pets, characterized by strong emotional attachment, anthropomorphism, and spoiling, often diminishes, as the need for pets to fill in the “child” role decreases.

Our results supported these expectations, although only the differences in Companionship and Your Affection reached at least a medium effect size, while the difference in Nurturance did not. Compared to owners with children, those without children rated their dogs higher on Instrumental Aid, Intimacy, Affection, and Your Antagonism scales (though these differences had small effect size), indicating somewhat stronger bonds and a higher tendency to anthropomorphize their pets^3^.

Contrary to the impact of parenthood, owner age did not notably influence dog-owner relationship ratings. Although small effect size differences indicated a trend for younger owners to rate their dogs higher on positive scales compared to older owners, these differences largely overlapped with those found between owners with and without children. This lack of significant age-related difference may be due to the small number of older participants in our sample or may align with the findings of El-Alayli et al.^62^, who observed no effect of participant characteristics (including age and duration of pet ownership) on pet-enhancement bias.

Regarding dog age, we expected owners of puppies to display more child-like relationship patterns compared to owners of adult dogs. However, neither the correlation with dog age nor the differences between puppies and adult dogs revealed any associations with at least medium effect size. This suggests that analyses can be conducted without controlling for the dog’s age, though further research involving more puppies is needed to confirm these findings.

Finally, we compared samples collected in 2011-13 and 2022-23. Similar to the above factors, the timing of the survey had minimal impact on the dog-owner relationship characteristics. Only two differences reached medium effect size: in 2022-23, romantic partners were rated higher in Instrumental Aid, and dogs in Reliable Alliance, compared to 2011-13. This confirms that the two samples can be merged. However, differences with small effect sizes suggest a trend: participants in 2022-23 reported receiving less support from their closest kin, best friend, and dog, but more from their romantic partner compared to 2011-13. The most likely explanation for these results is recent global events (e.g., COVID-19, war, inflation) and their negative effects on mental health^72,73^ and relationship quality^74,75^. Research on COVID-19 suggests that the pandemic significantly impacted social relationships, causing physical and emotional estrangement due to lockdown measures. This led to a decline in perceived social relationship quality and received social support^75^, likely affecting the dog-owner relationship as well. Interestingly, and contrary to our findings, a longitudinal study in the U.S. before, during, and after the pandemic^76^ showed a subtle increase in perceived emotional closeness to dogs. This indicates that while global events likely influenced the perceived quality of relationships, including those with dogs, cultural differences may also contribute to these variations, highlighting the complex nature of social relationships during times of crisis.

Nevertheless, our results show that although there is variability in the quality of the dog-owner relationship between individuals and over time, only a small part of this variability can be systematically explained by factors such as parenthood.

Three main limitations of the current study have already been mentioned:

1. Care should be taken when interpreting results related to the Reassurance of Worth scale due to its questionable reliability and relevance.
2. The study focused on the best or closest relationships, not the full social network. Participants selected one kin, one friend, and one child (if applicable), and one dog (if they had multiple). Results might differ if, instead of a single focal individual, we investigated the aggregate of each relationship type or a broader segment of the social network.
3. Like most research on the dog-owner bond, our participants were self-selected, predominantly female, and young to middle-aged, likely more satisfied with their relationships than the average dog owner. Aside from the fact that the sample likely lacks representation of dissatisfied owners, potentially painting a rosier picture of the dog-owner relationship, this introduces a bias in the results, as women and men tend to have different relationships with their dogs. For example, women tend to show greater empathy, stronger attachment to dogs, and are more likely to view them as family members or children compared to men (see reviews in^77,78^). Future research should complement the current findings by including a more diverse array of pet owners from different population types.

Another limitation is that far fewer participants rated their relationships with children compared to the other partners, which might have introduced a bias in the analysis. Moreover, while most hypotheses were set with pre-teen children in mind, this criterion was not enforced due to the limited number of parents in the sample. Future studies should differentiate between children of different ages.

Finally, since this study relied on subjective questionnaire assessments, it is unclear how well these results reflect reality. For instance, in real-life situations, owners might not prioritize their dogs over human partners. Additionally, as mentioned above, owners may interpret the relationship scales differently for dogs and human partners, highlighting the need for further research to develop more objectively comparable scales.

## Conclusions

In this study, we measured the social provisions provided by relationships with dogs and human partners to compare their roles in the social network and how they satisfy the different social needs of the owner. Our results indicate that the owner-dog relationship is most similar to the child-parent relationship but can overall be interpreted as a mix of child and best friend relationships, combining positive aspects of the child relationship with the lack of negative aspects of friendship, along with a unique level of relative power dynamics.

We found that human-dog relationships provide support through different forms of need fulfilment, predominantly via Companionship, Nurturance, and the absence of Negative Interactions. The support received from dogs was positively associated with support from human partners, contradicting the hypothesis that people acquire dogs to compensate for unsatisfied human support needs. However, support from dogs was rated higher than that from best friends and closest kin, which may suggest a positively distorted view of dogs by owners. Nonetheless, the dog-human relationship may indeed be the best some owners can get, offering features like unconditional love that may be harder to find in human partners.

All in all, confirming Bonas et al.^37^, we assert that characterizing the dog-owner relationship as a set of social provisions is a valid way to evaluate relationships with dogs. This concept offers an alternative theoretical framework, besides the attachment theory, that allows for the direct comparison of dog-human and human-human relationships. By linking human-animal bond research to the psychology of need fulfilment in human social relationships, this approach may provide a deeper understanding of the nature of the dog-human relationship and how it differs from human relationships.

## Electronic supplementary material

Below is the link to the electronic supplementary material.

Supplementary Material 1

Supplementary Material 2

## Data Availability

The dataset analyzed in the current study is available as supplementary material.
