# Supplementary material for: Similarities and differences between dog–human and human–human relationships
Source: Sci Rep. 2025 Apr 22;15:11871. doi: 10.1038/s41598-025-95515-8 (PMC12012045; doi:10.1038/s41598-025-95515-8)
Supplement: Supplementary file 2 — Supplementary Material 2 [file 41598_2025_95515_MOESM2_ESM.pdf]

Supplementary Information

Title: The role of the dog in human social networks: Comparing the relationship with a dog to human relationships

Authors: Borbála Turcsán, Dorottya Júlia Ujfalussy, Andrea Kerepesi, Ádám Miklósi, Enikő Kubinyi

Supplementary Table S1. Number of relationship assessments in the different data collection periods and in total, separately for each partner and each relationship scale.

Relationship scale	Partner	N of 2011-13	N of 2022-23	Total N
Companionship	closest kin	415	242	657
	child	0	112	112
	romantic partner	322	224	546
	best friend	409	246	655
	dog	429	282	711
Instrumental Aid	closest kin	411	245	656
	child	0	113	113
	romantic partner	320	230	550
	best friend	411	245	656
	dog	419	273	692
Intimacy	closest kin	412	245	657
	child	0	112	112
	romantic partner	319	228	547
	best friend	416	247	663
	dog	422	274	696
Nurturance	closest kin	412	244	656
	child	0	112	112
	romantic partner	318	222	540
	best friend	409	245	654
	dog	422	275	697
Affection (for participant)	closest kin	413	246	659
	child	0	113	113
	romantic partner	318	229	547
	best friend	414	246	660
	dog	420	280	700
Your Affection (for others)	sibling	418	246	664
	child	0	113	113
	romantic partner	318	227	545
	best friend	413	242	655
	dog	426	279	705
Reassurance of Worth	closest kin	415	244	659
	child	0	112	112
	romantic partner	317	229	546
	best friend	410	246	656
	dog	412	264	676
Reliable Alliance	closest kin	414	239	653

	child	0	112	112
	romantic partner	318	227	545
	best friend	416	242	658
	dog	427	273	700
Conflict	closest kin	414	242	656
	child	0	111	111
	romantic partner	317	228	545
	best friend	407	246	653
	dog	423	269	692
Antagonism (for participant)	closest kin	412	243	655
	child	0	111	111
	romantic partner	318	229	547
	best friend	415	247	662
	dog	421	276	697
Your Antagonism (for others)	closest kin	411	246	657
	child	0	113	113
	romantic partner	315	228	543
	best friend	411	249	660
	dog	420	281	701
Satisfaction	closest kin	416	240	656
	child	0	111	111
	romantic partner	319	228	547
	best friend	408	243	651
	dog	427	275	702
Relative Power	closest kin	405	240	645
	child	0	111	111
	romantic partner	314	226	540
	best friend	406	242	648
	dog	423	274	697

Supplementary Table S2. Results of the comparisons between the 2011-13 and 2022-23 samples in the ratings of the dog and of the three human partners common in the two samples (Mann-Whitney U test). Differences with at least medium effect size (|Cohen's d| > 5) are in bold. Positive values indicate that the dog received lower values in the 2022-23 sample than in the 2011-13 sample.

	Dog			Romantic partner			Best friend			Closest kin		
Relationship scales	z value	p value	Cohen's d (95% CI)	z value	p value	Cohen's d (95% CI)	z value	p value	Cohen's d (95% CI)	z value	p value	Cohen's d (95% CI)
SUPPORT	-1.543	0.123	0.112 (-0.041-0.265)	-4.008	< 0.001	-0.345 (-0.517--0.173)	-1.297	0.195	0.115 (-0.043-0.273)	-2.925	0.003	0.225 (0.066-0.384)
Companionship	-0.499	0.617	0.047 (-0.103-0.198)	-0.081	0.936	-0.021 (-0.191-0.150)	-3.359	0.001	0.267 (0.108-0.426)	-3.077	0.002	0.257 (0.098-0.416)
Instrumental Aid	-3.555	< 0.001	0.279 (0.125-0.432)	**-5.708**	**< 0.001**	**-0.506 (-0.678--0.334)**	-0.110	0.912	-0.019 (-0.177-0.139)	-1.160	0.246	0.071 (-0.087-0.229)
Intimacy	-4.004	< 0.001	0.309 (0.156-0.462)	-3.546	< 0.001	-0.325 (-0.496--0.154)	-1.108	0.268	0.073 (-0.084-0.231)	-2.924	0.003	0.252 (0.093-0.410)
Nurturance	-5.174	< 0.001	-0.329 (-0.482--0.176)	-3.117	0.002	-0.269 (-0.441--0.096)	-3.794	< 0.001	0.307 (0.148-0.466)	-2.976	0.003	0.232 (0.073-0.391)
Affection (for participant)	-3.408	0.001	0.236 (0.085-0.388)	-0.167	0.867	-0.030 (-0.199-0.140)	-4.179	< 0.001	0.329 (0.170-0.487)	-4.261	< 0.001	0.277 (0.118-0.435)
Your Affection (for others)	-3.575	< 0.001	0.254 (0.103-0.406)	-0.852	0.394	-0.105 (-0.275-0.065)	-2.679	0.007	0.240 (0.081-0.399)	-6.746	< 0.001	0.445 (0.286-0.605)
Reassurance of Worth	-0.718	0.473	-0.030 (-0.185-0.125)	-3.495	< 0.001	-0.325 (-0.496--0.154)	-0.602	0.547	-0.056 (-0.215-0.102)	-0.292	0.770	0.016 (-0.142-0.174)
Reliable Alliance	**-9.948**	**< 0.001**	**-0.637 (-0.792--0.481)**	-6.015	< 0.001	-0.447 (-0.619--0.274)	-5.170	< 0.001	-0.313 (-0.472--0.154)	-3.337	0.001	-0.076 (-0.235-0.084)
NEGATIVE INTERACTION	-3.329	0.001	-0.302 (-0.457--0.146)	-1.938	0.053	-0.175 (-0.347--0.002)	-1.493	0.135	0.060 (-0.100-0.220)	-0.017	0.987	-0.041 (-0.201-0.120)
Conflict	-1.452	0.147	-0.120 (-0.273-0.033)	-0.315	0.753	-0.013 (-0.183-0.157)	-1.299	0.194	0.091 (-0.067-0.249)	-0.336	0.737	0.029 (-0.130-0.187)
Antagonism (for participant)	-3.467	0.001	-0.379 (-0.532--0.226)	-3.963	< 0.001	-0.434 (-0.605--0.262)	-0.614	0.539	-0.090 (-0.248-0.067)	-2.321	0.020	-0.292 (-0.451--0.132)
Your Antagonism (for others)	-2.689	0.007	-0.218 (-0.369--0.066)	-0.060	0.952	-0.031 (-0.202-0.139)	-2.500	0.012	0.158 (0.000-0.315)	-3.126	0.002	0.178 (0.020-0.336)
Satisfaction	-0.023	0.982	-0.005 (-0.156-0.147)	-0.927	0.354	-0.071 (-0.241-0.099)	-1.918	0.055	-0.105 (-0.264-0.054)	-1.888	0.059	0.136 (-0.023-0.295)
Relative Power	-0.348	0.728	-0.080 (-0.232-0.072)	-1.336	0.182	0.121 (-0.050-0.293)	-0.534	0.593	0.053 (-0.106-0.212)	-0.083	0.934	-0.005 (-0.164-0.155)

Supplementary Table S3. Results of the pairwise comparisons between the ratings of the three blood kin partners on the 2011-13 sample (Wilcoxon signed rank test). The effect size of the difference and its 95% confidence interval is also provided. Negative values indicate that the first member of the pair received a lower value than the second member. Differences with at least medium effect size (|Cohen's d| > 5) are in bold.

	Mother vs. father				Mother vs. sibling				Father vs. sibling			
Relationship scales	N	z value	p value	Cohen's d (95% CI)	N	z value	p value	Cohen's d (95% CI)	N	z value	p value	Cohen's d (95% CI)
SUPPORT	**336**	**-10.490**	**< 0.001**	**0.602 (0.485-0.718)**	315	-6.875	< 0.001	0.416 (0.301-0.531)	292	-3.150	0.002	-0.241 (-0.357--0.124)
Companionship	**341**	**-9.470**	**< 0.001**	**0.553 (0.438-0.666)**	318	-1.427	0.154	0.107 (-0.003-0.217)	291	-6.450	< 0.001	-0.406 (-0.525--0.286)
Instrumental Aid	**340**	**-9.939**	**< 0.001**	**0.616 (0.500-0.732)**	310	-7.509	< 0.001	0.474 (0.356-0.591)	289	-1.416	0.157	-0.096 (-0.212-0.019)
Intimacy	**336**	**-11.289**	**< 0.001**	**0.720 (0.600-0.84)**	318	-5.036	< 0.001	0.296 (0.183-0.408)	292	-7.075	< 0.001	-0.436 (-0.556--0.316)
Nurturance	**336**	**-9.663**	**< 0.001**	**0.576 (0.460-0.691)**	314	-7.459	< 0.001	0.454 (0.337-0.569)	290	-3.103	0.002	-0.196 (-0.312--0.080)
Affection (for participant)	**341**	**-9.051**	**< 0.001**	**0.535 (0.421-0.648)**	**315**	**-9.236**	**< 0.001**	**0.589 (0.469-0.708)**	291	-0.542	0.588	0.016 (-0.099-0.131)
Your Affection (for others)	347	-7.984	< 0.001	0.455 (0.344-0.565)	319	-6.222	< 0.001	0.345 (0.232-0.458)	295	-2.348	0.019	-0.159 (-0.274--0.044)
Reassurance of Worth	340	-6.619	< 0.001	0.377 (0.267-0.487)	318	-3.937	< 0.001	0.238 (0.126-0.349)	290	-3.444	0.001	-0.206 (-0.322--0.090)
Reliable Alliance	339	-6.400	< 0.001	0.358 (0.248-0.467)	316	-4.413	< 0.001	0.219 (0.107-0.331)	292	-3.305	0.001	-0.202 (-0.318--0.086)
NEGATIVE INTERACTION	323	-4.028	< 0.001	0.212 (0.101-0.322)	292	-1.203	0.229	0.087 (-0.028-0.201)	272	-1.545	0.122	-0.076 (-0.195-0.043)
Conflict	338	-3.314	< 0.001	0.172 (0.064-0.279)	315	-1.751	0.080	0.100 (-0.010-0.211)	290	-0.363	0.716	-0.004 (-0.119-0.112)
Antagonism (for participant)	339	-1.951	0.051	0.092 (-0.015-0.198)	314	-1.206	0.228	0.068 (-0.042-0.179)	291	-0.215	0.829	0.019 (-0.096-0.134)
Your Antagonism (for others)	341	-6.022	< 0.001	0.341 (0.232-0.450)	315	-1.918	0.055	0.106 (-0.005-0.217)	291	-3.199	0.001	-0.185 (-0.301--0.069)
Satisfaction	341	-7.360	< 0.001	0.429 (0.317-0.539)	322	-4.410	< 0.001	0.249 (0.138-0.360)	293	-3.565	< 0.001	-0.228 (-0.344--0.112)
Relative Power	325	-1.540	0.124	0.087 (-0.022-0.196)	303	-2.398	0.017	-0.153 (-0.266--0.040)	278	-4.291	< 0.001	-0.266 (-0.386--0.147)

Supplementary Table S4. Results of the pairwise comparisons between the ratings of the dog and of the three human partners common in the two samples (Wilcoxon signed rank test) on the 2011-13 sample. The effect size of the difference and its 95% confidence interval is also provided. Negative values indicate that the dog received higher values than the human partner. Differences with at least medium effect size (|Cohen's d| > 5) are in bold.

	Closest kin				Romantic partner				Best friend			
Relationship scales	N	z value	p value	Cohen's d (95% CI)	N	z value	p value	Cohen's d (95% CI)	N	z value	p value	Cohen's d (95% CI)
SUPPORT	**404**	**-13.904**	**< 0.001**	**-0.821 (-0.933--0.708)**	310	-6.076	< 0.001	-0.382 (-0.497--0.267)	**404**	**-14.844**	**< 0.001**	**-0.975 (-1.093--0.856)**
Companionship	**411**	**-17.100**	**< 0.001**	**-1.704 (-1.855--1.552)**	**318**	**-11.223**	**< 0.001**	**-0.773 (-0.898--0.648)**	**406**	**-16.274**	**< 0.001**	**-1.417 (-1.555--1.279)**
Instrumental Aid	403	-2.882	0.004	-0.171 (-0.269--0.073)	310	-4.836	< 0.001	0.267 (0.153-0.380)	405	-4.478	< 0.001	-0.239 (-0.337--0.140)
Intimacy	405	-4.660	< 0.001	-0.243 (-0.341--0.144)	315	-6.936	< 0.001	0.409 (0.294-0.524)	410	-4.218	< 0.001	0.224 (0.125-0.321)
Nurturance	**406**	**-14.374**	**< 0.001**	**-0.968 (-1.086--0.85)**	**313**	**-8.502**	**< 0.001**	**-0.544 (-0.663--0.425)**	**406**	**-15.308**	**< 0.001**	**-1.142 (-1.266--1.016)**
Affection (for participant)	**404**	**-10.241**	**< 0.001**	**-0.563 (-0.668--0.458)**	311	-5.410	< 0.001	-0.307 (-0.421--0.193)	**405**	**-14.767**	**< 0.001**	**-1.060 (-1.181--0.938)**
Your Affection (for others)	**414**	**-11.764**	**< 0.001**	**-0.646 (-0.751--0.539)**	316	-7.084	< 0.001	-0.432 (-0.547--0.316)	**409**	**-15.659**	**< 0.001**	**-1.198 (-1.324--1.070)**
Reassurance of Worth	**399**	**-12.108**	**< 0.001**	**-0.738 (-0.848--0.627)**	308	-7.880	< 0.001	-0.496 (-0.614--0.377)	**397**	**-11.527**	**< 0.001**	**-0.679 (-0.788--0.570)**
Reliable Alliance	413	-7.937	< 0.001	-0.403 (-0.503--0.303)	**317**	**-11.962**	**< 0.001**	**-0.875 (-1.004--0.745)**	**415**	**-13.795**	**< 0.001**	**-0.903 (-1.016--0.788)**
NEGATIVE INTERACTION	**380**	**-11.759**	**< 0.001**	**0.676 (0.564-0.787)**	**288**	**-11.649**	**< 0.001**	**0.852 (0.716-0.986)**	375	-4.492	< 0.001	0.196 (0.093-0.298)
Conflict	**409**	**-11.833**	**< 0.001**	**0.675 (0.567-0.782)**	**313**	**-11.190**	**< 0.001**	**0.773 (0.646-0.898)**	402	-4.744	< 0.001	0.208 (0.109-0.307)
Antagonism (for participant)	403	-7.330	< 0.001	0.395 (0.293-0.496)	**311**	**-8.066**	**< 0.001**	**0.513 (0.395-0.631)**	408	-1.764	0.078	-0.092 (-0.189-0.006)
Your Antagonism (for others)	**403**	**-13.176**	**< 0.001**	**0.806 (0.693-0.918)**	**309**	**-13.249**	**< 0.001**	**1.103 (0.961-1.244)**	404	-8.418	< 0.001	0.452 (0.350-0.554)
Satisfaction	**413**	**-11.800**	**< 0.001**	**-0.666 (-0.772--0.559)**	**317**	**-8.426**	**< 0.001**	**-0.519 (-0.636--0.401)**	**406**	**-12.052**	**< 0.001**	**-0.740 (-0.849--0.630)**
Relative Power	**401**	**-14.824**	**< 0.001**	**-1.061 (-1.183--0.939)**	**309**	**-12.027**	**< 0.001**	**-0.901 (-1.033--0.768)**	**402**	**-14.749**	**< 0.001**	**-1.131 (-1.256--1.005)**

Supplementary Table S5. Results of the pairwise comparisons between the ratings of the dog and of the three human partners common in the two samples (Wilcoxon signed rank test) on the 2022-23 sample. The effect size of the difference and its 95% confidence interval is also provided. Negative values indicate that the dog received higher values than the human partner. Differences with at least medium effect size (|Cohen's d| > 5) are in bold.

	Closest kin				Romantic partner				Best friend			
Relationship scales	N	z value	p value	Cohen's d (95% CI)	N	z value	p value	Cohen's d (95% CI)	N	z value	p value	Cohen's d (95% CI)
SUPPORT	**236**	**-11.290**	**< 0.001**	**-0.947 (-1.100--0.793)**	219	-0.416	0.677	-0.024 (-0.156-0.108)	**241**	**-11.129**	**< 0.001**	**-0.934 (-1.085--0.782)**
Companionship	**242**	**-13.244**	**< 0.001**	**-2.030 (-2.250--1.809)**	**223**	**-8.512**	**< 0.001**	**-0.681 (-0.826--0.535)**	**246**	**-13.072**	**< 0.001**	**-1.698 (-1.893--1.502)**
Instrumental Aid	240	-0.657	0.511	0.016 (-0.111-0.142)	**225**	**-9.160**	**< 0.001**	**0.755 (0.607-0.903)**	242	-1.071	0.284	0.056 (-0.071-0.182)
Intimacy	241	-1.982	0.048	-0.139 (-0.265--0.012)	**222**	**-9.251**	**< 0.001**	**0.783 (0.632-0.933)**	244	-6.274	< 0.001	0.451 (0.319-0.582)
Nurturance	**242**	**-12.523**	**< 0.001**	**-1.335 (-1.508--1.161)**	**217**	**-7.401**	**< 0.001**	**-0.553 (-0.695--0.409)**	**243**	**-12.780**	**< 0.001**	**-1.515 (-1.699--1.330)**
Affection (for participant)	**245**	**-8.961**	**< 0.001**	**-0.673 (-0.812--0.534)**	229	-1.278	0.201	-0.089 (-0.219-0.041)	**245**	**-11.954**	**< 0.001**	**-1.163 (-1.325—1.000)**
Your Affection (for others)	**244**	**-10.997**	**< 0.001**	**-0.892 (-1.040--0.743)**	225	-1.631	0.103	-0.138 (-0.269--0.006)	**241**	**-12.038**	**< 0.001**	**-1.222 (-1.388--1.054)**
Reassurance of Worth	**233**	**-9.326**	**< 0.001**	**-0.763 (-0.908--0.616)**	217	-3.577	< 0.001	-0.252 (-0.387--0.117)	**235**	**-7.498**	**< 0.001**	**-0.541 (-0.677--0.403)**
Reliable Alliance	**238**	**-7.734**	**< 0.001**	**-0.541 (-0.677--0.405)**	**225**	**-9.152**	**< 0.001**	**-0.721 (-0.867--0.574)**	**241**	**-9.930**	**< 0.001**	**-0.768 (-0.911--0.623)**
NEGATIVE INTERACTION	232	-6.676	< 0.001	0.462 (0.326-0.597)	**216**	**-10.345**	**< 0.001**	**0.915 (0.755-1.073)**	237	-2.156	0.031	-0.159 (-0.287--0.031)
Conflict	**235**	**-7.565**	**< 0.001**	**0.555 (0.417-0.691)**	**221**	**-9.327**	**< 0.001**	**0.771 (0.62-0.921)**	239	-0.531	0.596	0.007 (-0.119-0.134)
Antagonism (for participant)	239	-3.683	< 0.001	0.250 (0.121-0.378)	**225**	**-7.386**	**< 0.001**	**0.563 (0.421-0.703)**	244	-5.405	< 0.001	-0.340 (-0.469--0.211)
Your Antagonism (for others)	246	-6.750	< 0.001	0.457 (0.325-0.588)	**227**	**-11.144**	**< 0.001**	**1.099 (0.933-1.263)**	249	-0.642	0.521	0.037 (-0.087-0.161)
Satisfaction	**237**	**-9.891**	**< 0.001**	**-0.786 (-0.931--0.640)**	227	-5.825	< 0.001	-0.431 (-0.566--0.294)	**240**	**-8.433**	**< 0.001**	**-0.625 (-0.762--0.486)**
Relative Power	**239**	**-12.046**	**< 0.001**	**-1.265 (-1.435--1.094)**	**223**	**-11.736**	**< 0.001**	**-1.255 (-1.429--1.078)**	**241**	**-12.542**	**< 0.001**	**-1.490 (-1.673--1.306)**

Supplementary Table 6. Results of the pairwise comparisons of the distance to the dog index between the four human partners (Wilcoxon signed rank test) separately in the two samples. The effect size of the difference and its 95% confidence interval is also provided. Negative values indicate that the first member of the pair has a smaller distance index value (is more similar to the dog) than the second member. Distance indexes were calculated from all scales (global), only positive scales (support), and only negative scales (negative interaction). Differences with at least medium effect size (|Cohen's d| > 5) are in bold.

	GLOBAL (all scales)				SUPPORT				NEGATIVE INTERACTION			
Pairs	N	z value	p value	Cohen's d (95% CI)	N	z value	p value	Cohen's d (95% CI)	N	-z value	p value	Cohen's d (95% CI)
2011-2013 sample												
Romantic partner vs. best friend	266	-3.153	0.002	-0.144 (-0.265--0.023)	300	-5.677	< 0.001	-0.300 (-0.415--0.184)	**274**	**-8.302**	**< 0.001**	**0.539 (0.412-0.665)**
Romantic partner vs. closest kin	268	-3.968	< 0.001	0.265 (0.143-0.386)	301	-5.246	< 0.001	0.335 (0.219-0.451)	275	-3.151	0.002	-0.173 (-0.292--0.054)
Best friend vs. closest kin	353	-2.346	0.019	0.197 (0.092-0.303)	394	-0.855	0.393	0.134 (0.035-0.233)	364	-6.146	< 0.001	0.342 (0.237-0.448)
2022-2023 sample												
Romantic partner vs. best friend	194	-2.361	0.018	-0.193 (-0.335--0.051)	202	-4.45	< 0.001	-0.346 (-0.488--0.204)	198	-4.729	< 0.001	0.352 (0.208-0.495)
Romantic partner vs. closest kin	189	-3.738	< 0.001	0.312 (0.166-0.458)	199	-4.435	< 0.001	0.371 (0.227-0.515)	194	-2.81	0.005	-0.192 (-0.334--0.050)
Best friend vs. closest kin	219	-1.554	0.120	0.199 (0.065-0.333)	229	-0.468	0.639	0.146 (0.016-0.276)	222	-2.582	0.010	0.190 (0.057-0.323)
